# Geographic and genetic diversity in gallbladder cancer mutation profiles: insights from a worldwide exome analysis

**DOI:** 10.1016/j.ebiom.2026.106305

**Published:** 2026-05-22

**Authors:** Valentina Gárate-Calderón, Rajiv Kumar, Katherine Marcelain, Linda Zollner, Felix Boekstegers, Olga Barajas, Denisse Loader, María Teresa Rivera, Erik Morales, Gonzalo de Toro, Christian Caglevic, Tatsuhiro Shibata, Justo Lorenzo Bermejo

**Affiliations:** a Statistical Genetics Research Group, Institute of Medical Biometry, Heidelberg University, Heidelberg, Germany; b Department of Basic and Clinical Oncology and Center for Cancer Prevention and Control (CECAN), Faculty of Medicine, Universidad de Chile, Santiago, Chile; c Hospital Padre Hurtado, Santiago, Chile; d Servicio de Anatomía Patológica, Hospital del Salvador, Santiago, Chile; e Facultad de Medicina, Universidad Católica del Maule and Hospital Regional de Talca, Talca, Chile; f Hospital de Puerto Montt, Puerto Montt, Chile; g Cancer Research Department, Instituto Oncológico Fundación Arturo Lopez Perez, Santiago, Chile; h Division of Cancer Genomics, National Cancer Center Research Institute, Tokyo, Japan; i Laboratory of Molecular Medicine, Human Genome Center, Institute of Medical Science, The University of Tokyo, Tokyo; j Laboratory of Biostatistics for Precision Oncology, Institut Strauss, Strasbourg, France

**Keywords:** Gallbladder cancer, Whole-exome sequencing, Somatic mutations, Mutational signatures, Genetic ancestry, Cancer genomics

## Abstract

**Background:**

Gallbladder cancer (GBC), an aggressive disease with limited treatment options, occurs mainly in low-income and middle-income regions of Asia and Latin America. We analysed whole-exome sequencing data from 262 GBC tumour–normal sample pairs from Chilean, Chinese, Indian, Japanese and South Korean patients to investigate differences in GBC mutation profiles according to geographic location and genetic ancestry.

**Methods:**

We used a unified analysis pipeline to ascertain gene mutations and mutational signatures, estimated the individual proportions of major ancestry subtypes in the investigated cohorts, and examined the relationship between genetic and genomic profiles.

**Findings:**

The tumour mutation burden (TMB) was highest in China and lowest in Chile (p < 0.0001 [F-test]). The most frequently mutated genes included *TP53, MUC16, ELF3,* and *ARID2*. The most common mutational signatures were single-base substitution (SBS)5, SBS1, and SBS13. We observed differences between cohorts in the frequency of specific gene mutations and mutational signatures, as well as associations between the major ancestry subtypes in the investigated cohorts and (1) TMB, (2) the frequencies of *MACF1* and *MUC16* mutations, and (3) the frequencies of SBS5, SBS13, and SBS29. For example, TMB increased by 0.08 (95%CI 0.03–0.14) for every 1% increase in the proportion of Japanese ancestry.

**Interpretation:**

GBC exhibits marked geographic and genetic heterogeneity in its mutation profiles, which could have relevant implications for prevention and targeted therapy in the high-incidence regions investigated.

**Funding:**

This study was supported by the 10.13039/501100007601European Union’s Horizon 2020 research and innovation programme (grant 825741) and the German Academic Exchange Service (DAAD; grant 91762082).

**Research in context**

**Evidence before this study**

Previous whole-exome sequencing studies have shown that gallbladder cancer is molecularly heterogeneous, with recurrent alterations in *TP53*, genes in the ERBB pathway, chromatin-remodelling genes, and specific mutational signatures. However, the available evidence remains scarce and derives mainly from single-country cohorts, analysed using distinct protocols, which hinders direct comparison between populations. Harmonised whole-exome analyses of matched tumour–normal samples from high-incidence populations in Asia and Latin America have been limited, and the contribution of genetic ancestry to the variation in mutation characteristics remains largely unexplored.

**Added value of this study**

We applied a harmonised analytical pipeline to whole-exome sequencing data from 262 paired tumour–normal samples from Chilean, Chinese, Indian, Japanese, and South Korean patients with gallbladder cancer. *TP53, MUC16, ELF3*, and *ARID2* were the most frequently mutated genes, and SBS5, SBS1, and SBS13 were the most common mutational signatures. The cohorts differed markedly in tumour mutation burden, and in the frequency of specific gene mutations and mutational signatures. We also identified associations between particular ancestry components and specific gene mutations and mutational signatures.

**Implications of all the available evidence**

Taken together, the unified analysis of whole-exome sequencing data from multiple cohorts suggests that gallbladder cancer exhibits a heterogeneous mutation profile across different populations. This finding has implications for biomarker discovery, molecular stratification, and the design of translational studies in regions where the burden of the disease is highest. Future studies should validate the identified associations between genetic ancestry and gallbladder cancer genomics, integrating data on exposure, pathology, treatment, and patient outcomes in larger, geographically diverse cohorts.

## Introduction

Gallbladder cancer (GBC; diagnosis code C23 in the International Classification of Diseases, 10th Revision) is an aggressive tumour that forms in the gallbladder. The most important risk factors for the development of GBC include the presence of gallstones, which can cause inflammation of the gallbladder; age and female sex; overweight and obesity; a family history of GBC; recently identified susceptibility variants in the Indian population, validated in the Chilean population as well; and, in Latin Americans, the individual proportion of Indigenous American ancestry.^1^, ^2^, ^3^, ^4^, ^5^ Early GBC symptoms are non-specific and may include abdominal bloating and pain, usually in the upper right region, weight loss and jaundice. As a result, most GBC tumours are characterised by rapid progression and a poor prognosis.

Epidemiological studies have shown substantial geographic and ethnic differences in the incidence of GBC, with the highest rates observed in certain countries of South Asia, including Bangladesh, India and Nepal, East Asia (South Korea, Japan and China), and Latin America, particularly Bolivia, Chile, southern Peru and north-western Argentina.^6^, ^7^, ^8^ Asian cohorts of GBC patients have shown a large diversity in the molecular processes underlying tumour development, with multiple driver genes affected and heterogeneous mutational signatures identified.^9^, ^10^, ^11^, ^12^, ^13^ For example, Nakamura et al. identified novel genomic alterations and potential therapeutic targets in biliary tract cancers, revealing subtype-specific mutations and the potential for immunomodulatory therapies, especially for patients with hypermutated tumours and poor prognosis.^9^ Wardell et al. applied large-scale genomic sequencing to unveil distinct somatic and germline mutations in biliary tract cancers, offering insights into their origins, classification and potential for personalised treatment.^11^ Pandey et al. identified new and targetable genetic mutations and pathways, including potential GBC vaccine candidates, offering new therapeutic opportunities for this aggressive disease.^13^ The study by Nepal et al. revealed three molecular subtypes of GBC linked to patient survival, demonstrating that tumour microenvironment and immune features, along with genetic mutations, play key roles in gallbladder carcinogenesis and prognosis.^12^ While these studies have included a few Chilean patients, molecular characterisation of GBC in Chile has so far focused on tumour DNA methylation.^14^

Disparities in incidence across geographic regions suggest that environmental and genetic factors contributing to the risk of GBC may vary according to specific population characteristics. However, the mechanisms that cause these disparities remain poorly understood.^15^ Due to the relatively low incidence of GBC in high-income regions, research into GBC has been largely neglected compared with other tumours such as bile duct cancer.^6^ GBC can be considered an understudied disease, with suboptimal prevention, diagnosis and treatment strategies.^8^ The poor scientific evidence available limits the ability to improve the control of this devastating malignancy, particularly in low-income and middle-income regions of high incidence.

We analysed whole-exome sequencing data from 262 pairs of tumour and normal samples from Chilean, Chinese, Indian, Japanese and South Korean patients with GBC to investigate possible geographic and genetic differences in mutation profiles. We applied a unified analysis pipeline to determine gene mutations and mutational signatures, estimated the proportions of major ancestry subtypes in the patient cohorts, and examined the relationship between the genetic profiles of the patients and the genomic profiles of their tumours.

## Methods

### Investigated datasets

We compiled whole-exome sequencing data from 249 GBC tumour–normal sample pairs from previously published Chilean, Chinese, Indian, Japanese and South Korean patients and from 13 additional Chilean patients with GBC, totalling 262 GBC samples.^9^^,^^11^, ^12^, ^13^ Formalin-fixed paraffin-embedded (FFPE) tumour tissue, blood samples and clinical information from the 13 new Chilean patients were collected after written informed consent, complying with the ethical guidelines of the 1975 Declaration of Helsinki. DNA was extracted from FFPE sections and whole blood using the Qiagen kits AllPrep DNA/RNA FFPE and QIAamp DNA Blood, respectively. DNA quality was controlled, and exome capture was carried out using the SureSelect XT Human All Exon V6 Plus library (Agilent Technologies). The kits used for exome capture in the investigated cohorts are shown in Supplementary Table S1. Sex information was self-reported by study participants and included as a covariate in the regression models.

### Ethics

The study protocol for analysing 13 Chilean GBC FFPE and blood samples was reviewed and approved by the appropriate ethics committees in Chile. Patients were recruited at Hospital del Salvador (#06.10.2015 CEC SSMO), Instituto Nacional del Cancer (approval #08.03.2016 CEC SSMO), Hospital Padre Hurtado (#15.10.2015 CEC SSMSO), Hospital Regional de Talca (#08.01.2019 CEC SSMO) and Hospital de Puerto Montt (26.07.2016 CEC SSMO), as well as the Biobank of Universidad de Chile (#123-2012). All participants provided written informed consent prior to enrolment in the study and sample collection.

### Genetic ancestry analysis

Whole-exome sequencing reads of the normal samples were mapped to the human reference genome (GRCh38), and the germline variants of the patients with GBC were called with the HaplotypeCaller according to GATK best practices to generate gVCFs.^16^ We then excluded non-autosomal variants and polymorphisms with missing call rates above 5% or minor allele frequencies below 5%.

The remaining 75,894 variants were combined with single-nucleotide polymorphism data from the 1000 Genomes Project (1000 GP) and Human Genome Diversity Project (HGDP) populations, eliminating closely related individuals and low-quality Oceanian samples (3402 individuals retained for further analysis), to perform principal component analysis (PCA) and estimate individual proportions of genetic ancestry.^17^

To identify the major ancestry subtypes in the investigated cohorts, we first performed a supervised ADMIXTURE analysis using 1000 GP and HGDP subpopulations defined by geographic/genetic region labels (AFR = African, AMR = Admixed American, CSA = Central/South Asian, EAS = East Asian, EUR = European, MID = Middle Eastern). We then carried out an unsupervised ADMIXTURE analysis to select the main ancestry subtypes, considering the clustering of GBC patient cohorts and reference subpopulations observed in the PCA. To this end, a cross-validation was performed to determine the number of subpopulations that minimised variance. We considered all subpopulations associated with each geographic/genetic region label, and selected those that together explained more than 40% of the variability, with each subpopulation contributing at least 10%. The selected populations were then used to examine the relationship between the type and proportion of genetic ancestry and GBC mutation profiles.

The Chilean genome results from genetic admixture between Indigenous Americans, Europeans and Africans.^18^ On this basis, we used as reference populations for estimating ancestry in the Chilean GBC patients a subset of 16 Peruvians in Lima from the 1000 GP with 100% Indigenous American ancestry according to unsupervised ADMIXTURE results (hereafter referred to as Pel-AMR), the Iberian populations in Spain (IBS) and the Yoruba in Ibadan, Nigeria (YRI) from the 1000 GP. PCAs were performed using Eigenstrat,^19^ and ADMIXTURE version 1.3 was used for ancestry estimation.^20^

### Somatic SNV and indel calling and annotation

Paired-end reads from tumour and matched normal samples were aligned to the human reference genome (GRCh38) using Burrows–Wheeler Aligner.^21^ Read quality was assessed with FastQC (v0.11.9), and duplicate reads were marked with Picard (v3.1.1). To harmonise analyses across cohorts, we defined a shared exome region as the intersection of the five cohort-specific capture interval sets after coordinate normalisation, sorting, and merging. This shared region spanned 32.12 Mb and was used as the denominator to calculate the tumour mutation burden (TMB) and other coverage metrics.

Somatic single-nucleotide variants (SNVs) and small insertions and deletions (indels) were called independently with Mutect2 (GATK v4.2.2.0),^16^ and VarScan2 somatic (v2.4.6)^22^ in paired tumour–normal mode. Mutect2 calls were filtered according to GATK Best Practices, including hard filtering, orientation-bias filtering and a minimum tumour depth of 30 reads. To improve comparability across cohorts and reduce caller-specific artefacts, we retained a harmonised consensus callset comprising variants identified by both callers within the shared exome region. Variants were annotated with ANNOVAR,^23^ and exonic and splice-site variants were retained for downstream analyses. Gene-based analyses and TMB calculations were restricted to non-synonymous exonic and splice-site variants.

### Mutational signature analysis

Mutational signature analysis was performed with SigProfilerAssignment^24^ using high-confidence somatic SNVs identified by Mutect2 after filtering. Variants were not restricted to the shared exome region and did not require concordance with VarScan2, because we aimed to capture mutational patterns at the sample level rather than derive a conservative consensus callset for gene-based comparisons. We report all COSMIC single-base substitution (SBS) signatures assigned at the sample level, together with the cosine similarity between the observed and reconstructed mutational profiles. Cohort-level cosine similarity summaries are provided in Supplementary Table S2, and sample-level assignment metrics, including the COSMIC SBS signatures identified in each tumour, are provided in Supplementary Table S3.

### Identification of recurrent gene mutations and copy-number analysis

Recurrent gene mutations were identified using MutSig2CV (v3.11),^25^ with a threshold of q ≤ 0.10 as the criterion for statistical significance. For cross-cohort analyses, we selected 28 genes combining MutSig2CV-q values with the identification of gene mutations in ≥10 samples, and we also included six previously reported gallbladder cancer driver genes (*BRCA1, BRCA2, BRAF, KRAS, NF1*, and *PIK3CA*).^26^, ^27^, ^28^

Allele-specific copy-number alterations were inferred from matched tumour–normal data with FACETS (v0.6.2)^29^ within the shared exome region. The burden of copy-number alterations was quantified as the proportion of base pairs in the exome region shared by all cohorts with copy number alterations; hereinafter referred to as the fraction of genome altered (FGA). GISTIC2 (v2.0.23)^30^ was applied separately to each cohort using FACETS-derived segmented copy-number profiles, restricted to autosomes and harmonised to GRCh38/Hg38. Significant lesions were defined at q < 0.25. Given the relatively small size of some cohorts, peak detection was interpreted descriptively for cross-cohort comparisons, rather than as definitive evidence for the presence of specific driver copy-number alterations.

### Coverage assessment and visualisation

Coverage metrics were calculated over the shared exome region using Picard CollectHsMetrics to assess sequencing performance across cohorts. An oncoplot was generated in R software environment for statistical computing and graphics (v4.0.3), using the Maftools package (v2.18.0).

### Statistical analyses

Statistical analyses were performed to assess differences in mutation characteristics across cohorts, as well as their association with the main subtypes of ancestry in the investigated cohorts. Linear regression models were fitted using TMB, or FGA, as the response variable. Logistic regression models were fitted using the presence of specific gene mutations, or specific mutational signatures, as the response variable. Depending on the analysis, the study cohort or the proportion of individual ancestry was included as the main explanatory variable, together with age at diagnosis grouped into quartiles, sex, gallstones, tumour stage (2 categories according to the American Joint Committee on Cancer [AJCC]), and the coverage in tumour and normal tissues as potential confounding factors. Frequencies and odds ratios (OR) are reported with 95% confidence intervals (CIs). Regression analyses were conducted using SAS version 9.4 (SAS Institute Inc), and graphs were created using the R software environment for statistical computing and graphics (version 4.0.3).

### External reference

The *Gallbladder Carcinoma* (MSK, 2022)^31^ cohort from cBioPortal was used as an external reference. It was not included in the harmonised analysis of whole-exome sequencing data because this dataset was generated using the MSK-IMPACT targeted sequencing platform, included both primary and metastatic tumours, and used a different variant annotation framework. Instead, we used it as a descriptive comparator in the discussion section for selected clinically relevant alterations.

### Role of funders

The funders had no role in study design, data collection, data analyses, interpretation, or writing of the report.

## Results

### Genetic ancestry of GBC patients

To identify the major types of ancestry in the investigated cohorts, we combined single-nucleotide polymorphism data from the five cohorts, the 1000 Genomes Project (1000 GP) and the Human Genome Diversity Project (HGDP), performed genetic PCAs, and estimated the individual proportions of genetic ancestry of GBC patients using ADMIXTURE.

Supplementary Fig. S1A shows the geographic distribution of the gallbladder cancer (GBC) cohorts included in this study, while Supplementary Fig. S1B depicts the nine reference subpopulations used for ancestry inference. These included IBS and Pel-AMR as proxies for European and Indigenous American ancestry, four reference groups from eastern China, two from northern India, and one Japanese reference group from Honshu. PCA plots confirmed that the Chilean patients were clustered with the admixed American populations in 1000 GP and HGDP, the Chinese, Japanese and South Korean patients with the East Asian populations, and the Indian GBC patients with the Central/South Asian populations (Supplementary Fig. S2).

**Fig. 1:**
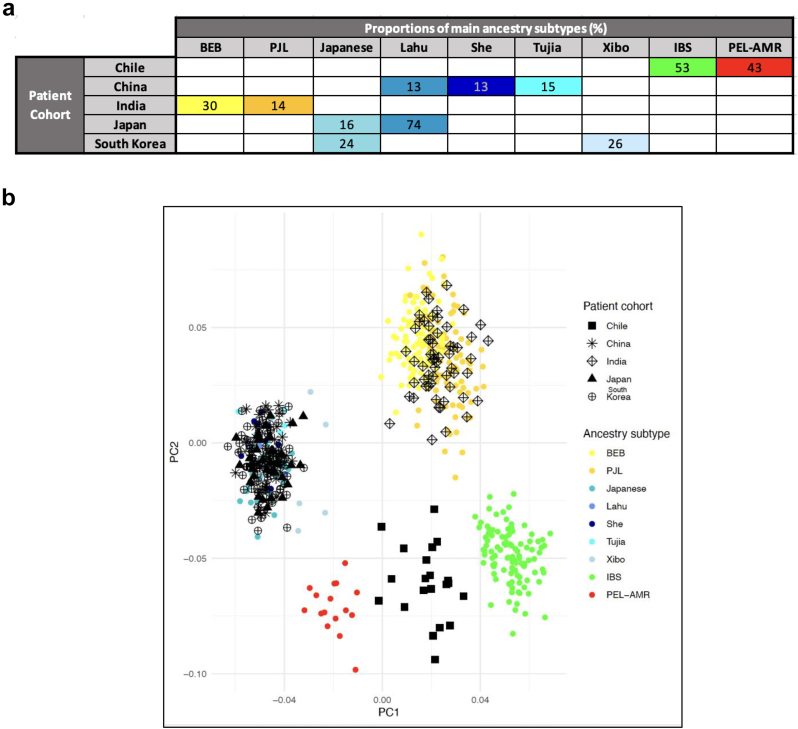
**Genetic ancestry of the investigated cohorts of gallbladder cancer patients.** (A) Average proportions of the major genetic ancestry subtypes in the five investigated patient cohorts. (B) Genetic principal component analysis (PCA) of the five investigated cohorts and the nine reference panels used to estimate the proportions of genetic ancestry. BEB, Bengali in Bangladesh (1000 Genomes Project, 1000 GP); PJL, Punjabi in Lahore, Pakistan (1000 GP); Japanese, Japanese population (Human Genome Diversity Project, HGDP); Lahu, Lahu population (HGDP); She, She population (HGDP); Tujia, Tujia population (HGDP); Xibo, Xibo population (HGDP); IBS, Iberian population in Spain (1000 GP); Pel-AMR, Peruvians from Lima with estimated 100% Indigenous American ancestry (1000 GP); PC1: First principal component; PC2: Second principal component.

The average proportions of the major ancestry subtypes across the investigated cohorts are summarised in Fig. 1A. Supervised and unsupervised results of ADMIXTURE showed that the main subtypes of genetic ancestry in the Chilean cohort were European–Iberian (the average proportion of ancestry attributed to Iberian populations in Spain [IBS] was 53%) and Indigenous American (43% average proportion attributed to Peruvian in Lima with estimated 100% Indigenous American ancestry [Pel-AMR]; Supplementary Figs. S3 and S4). The major subtypes of genetic ancestry in the Chinese cohort encompassed Tujia (15% average ancestry proportion), She (13%), and Lahu (13%); other reference subpopulations in 1000 GP and HGDP contributed with less than 10% ancestry (Supplementary Fig. S5). The main components of genetic ancestry in Indian GBC patients were Bengali in Bangladesh (BEB, 30% average ancestry proportion) and Punjabi in Lahore (PJL, 14%, Supplementary Fig. S6). Interestingly, the main subtype of ancestry in the Japanese cohort was Lahu (74%), followed by the Japanese subpopulation in HGDP (16%, Supplementary Fig. S7). The Japanese subpopulation 1000 GP contributed only 7% and was therefore not included in the subsequent analyses. The major ancestry subtypes in the South Korean cohort were Xibo (26%) and Japanese (24%, Supplementary Fig. S8). Two reference subpopulations in the HGDP contributed to multiple cohorts: the Lahu subpopulation contributed to the Chinese and Japanese cohorts, while the Japanese subpopulation contributed to the South Korean and Japanese cohorts.

Next, we used the nine major ancestry subtypes for supervised ADMIXTURE analyses and PCA plots. Fig. 1B shows that Chilean GBC patients had an admixed IBS/Pel_AMR ancestry, the Indian cohort overlapped with the BEB and PJL subpopulations, and the Chinese, Japanese, and South Korean cohorts clustered with the Japanese, Lahu, She, Tujia and Xibo subpopulations. The first principal component explained about 5.5% of the genetic variability and separated the East and the Central/South Asian, as well as the Indigenous American and European ancestry components. The second principal component explained about 4.0% of the variability and distinguished between Asians and non-Asians. Separate PCA plots for the three East Asian cohorts are shown in Supplementary Fig. S9.

### Gene mutations and mutational signatures of GBC tumours

To characterise the mutation landscape of GBC, we applied a unified data analysis pipeline to worldwide whole-exome sequencing data from 262 tumour–normal sample pairs. The median age at GBC diagnosis was 54 years in the investigated cohorts, and 64% of the patients were women (Table 1). Most of the GBC tumours studied were from South Korea (35%), followed by India (23%), China (19%), Japan (14%) and Chile (9%).

**Table 1:** Demographic and clinical characteristics of the study population.

Variable	Level	n	%
Age (years)	Less than 54	57	22
	54–62	61	23
	63–70	57	22
	71 or more	62	24
	Missing	25	9
Sex	Female	167	64
	Male	91	35
	Missing	4	1
Tumour stage (AJCC staging)	I–II	103	39
	III–IV	136	52
	Missing	23	9
Gallstone history	Positive	63	24
	Negative	9	3
	Missing	190	73
Type of normal sample	Blood	190	73
	Adjacent normal tissue	72	27
Type of tumour sample	Fresh-frozen	249	95
	FFPE	13	5
Patient cohort	Chile	23	9
	China	51	19
	India	59	23
	Japan	38	14
	South Korea	91	35

AJCC, American Joint Committee on Cancer; FFPE, Formalin-fixed, paraffin-embedded.

After harmonisation of whole-exome sequencing data, we identified 41,531 nonsynonymous somatic variants.

The TMB differed between cohorts (p < 0.0001 [Type III partial F-test]), median TMB 7.88 in the Chinese compared to 1.56 in the Chilean cohort (Table 2). The most frequently mutated genes were *TP53* (37%), *MUC16* (22%), *ELF3* (17%), *ARID2* (14%), *ERBB2* (11%), and *ARID1A* (10%). Other recurrently altered genes included *CTNNB1* and *ERBB3* (8% each), *SMAD4* (6%), *BRCA2* (6%), *PIK3CA* (6%) and *NF1* (5%). MutSig2CV showed strongest statistical support for recurrent mutation in *TP53, CDKN2A, ARID2, ERBB2, SMAD4, KRAS, ERBB3, CTNNB1,* and *ARID1A* (Table 3).

**Table 2:** Differences between cohorts in tumour mutation burden (TMB) and the fraction of base pairs with copy number alterations (Fraction of genome altered - FGA).

Genomic feature	Cohort	N	Median (IQR)	p value [F-test]
TMB	Chile	23	1.56 (0.93–2.52)	<0.0001
	China	51	7.88 (6.79–9.90)	
	India	59	1.62 (0.40–2.46)	
	Japan	38	3.75 (1.56–6.91)	
	South Korea	91	1.96 (1.28–3.14)	
FGA	Chile	23	0.34 (0.16–0.72)	0.04
	China	51	0.10 (0.02–0.14)	
	India	59	0.28 (0.00–0.55)	
	Japan	38	0.25 (0.10–0.69)	
	South Korea	91	0.29 (0.14–0.72)	

IQR, interquartile range; p value, probability value from a multivariable linear regression model that included study cohort, age, sex, gallstones, tumour stage, and coverage in tumour and normal tissues as explanatory variables.

**Table 3:** Mutation frequencies in 34 genes, and frequencies of recurrent mutational signatures.

Gene or mutational signature	n	%	MutSig2CV q value
*TP53*	97	37	9.4 × 10^−13^
*MUC16*	57	22	0.049
*ELF3*	45	17	9.4 × 10^−13^
*ARID2*	37	14	1.1 × 10^−5^
*ERBB2*	28	11	1.9 × 10^−5^
*ARID1A*	26	10	0.015
*MACF1*	25	10	0.069
*APOB*	23	9	0.044
*CTNNB1*	20	8	0.008
*ERBB3*	20	8	0.009
*FCGBP*	18	7	0.044
*WDR87*	18	7	0.049
*SMAD4*	17	6	9.1 × 10^−5^
*BRCA2*	16	6	1
*TCHH*	16	6	0.047
*FLG*	15	6	0.031
*PIK3CA*	15	6	1
*ZBED6*	14	5	0.049
*NF1*	13	5	1
*PCDH11X*	13	5	0.038
*ACAN*	12	5	0.076
*ANKZF1*	12	5	0.004
*DNMBP*	12	5	0.047
*CCNB3*	11	4	0.044
*IGSF1*	11	4	0.030
*MTOR*	11	4	0.076
*TBC1D8*	11	4	0.044
*VPS13A*	11	4	0.047
*CDKN2A*	10	4	3.9 × 10^−10^
*GDPD4*	10	4	0.025
*IL1*1RA	10	4	1.1 × 10^−4^
*KRAS*	8	3	2.3 × 10^−4^
*BRCA1*	5	2	1
*BRAF*	2	1	1
SBS5	192	73	NA
SBS1	148	56	NA
SBS13	104	40	NA
SBS2	87	33	NA
SBS29	47	18	NA
SBS7a	33	13	NA
SBS15	31	12	NA
SBS42	30	11	NA
SBS10b	29	11	NA
SBS39	27	10	NA

Frequencies are shown for 28 genes with the highest mutation frequencies in the study population, six previously reported gallbladder cancer driver genes (*BRCA1, BRCA2, BRAF, KRAS, NF1*, and *PIK3CA*) and the mutational signatures detected in at least 20 tumours. Low q values (calculated using MutSig2CV) indicate a significant increase in mutation frequency, taking into account the size of the gene. NA, not applicable.

Mutational signature assignment identified several recurrent COSMIC SBS signatures. Eleven signatures were detected in at least 20 tumours, with SBS5 (73%) and SBS1 (56%) being the most frequent, followed by SBS13 (40%), SBS2 (33%), and SBS29 (18%). Additional recurrent signatures included SBS7a (13%), SBS15 (12%), SBS42 (11%), SBS10b (11%), and SBS39 (10%) (Table 3). Cohort-specific mutational signature profiles are shown in Supplementary Figs. S10–S14. To provide a complete overview beyond the recurrent signatures highlighted in Table 3, we additionally report all COSMIC SBS signatures assigned at the sample level in Supplementary Table S3. Because signature assignment was based on filtered Mutect2 SNVs, we summarised the cosine similarity between observed and reconstructed mutational profiles separately from the consensus somatic variant analyses; these cohort-level summaries are shown in Supplementary Table S2. Together, these supplementary data allow assessment of both the range of signature assignments and the goodness-of-fit of the mutational signature reconstruction across cohorts.

**Fig. 2:**
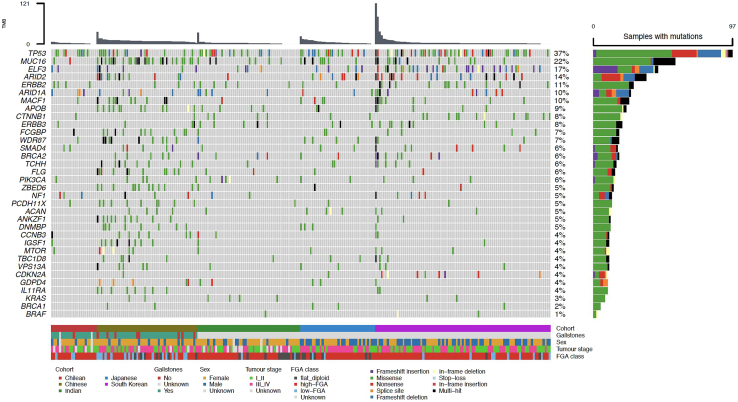
**Overview of somatic alterations in gallbladder cancer.** Oncoplot showing somatic mutations in 34 genes across 262 tumours from five cohorts, including the 28 genes with the highest mutation frequencies in the study population and six previously reported gallbladder cancer driver genes (*BRCA1, BRCA2, BRAF, KRAS, NF1*, and *PIK3CA*). The top panel shows the tumour mutation burden (TMB) and the right-hand panel shows the mutation frequency. Clinical and genomic features shown at the bottom include cohort, gallstones, sex, tumour stage, and FGA class, defined according to the fraction of genome altered (FGA) as flat_diploid (FGA = 0), low-FGA (0 < FGA < 0.10), or high-FGA (FGA ≥ 0.10).

The distribution of recurrent gene mutations, mutational signatures, and copy-number alteration categories is represented in Fig. 2.

### Copy-number alterations and their relationship with tumour mutation burden

To further characterise genomic alterations beyond gene mutations, we calculated the FGA as a measure of the whole-exome copy-number deviation from the diploid state. The distribution of FGA varied across cohorts, with a median FGA ranging from 0.10 in the Chinese cohort to 0.34 in the Chilean cohort, and intermediate values observed in India, Japan, and South Korea (p = 0.04 [F-test], Table 2). To compare copy-number landscapes across cohorts, we generated cohort-specific heatmaps and GISTIC2 summaries based on FACETS-derived segmented profiles. These analyses revealed substantial heterogeneity in copy-number alteration patterns across cohorts, affecting both broad chromosomal alterations and recurrent focal events (Supplementary Figs. S15 and S19). The Chinese cohort showed a relatively low copy-number burden, consistent with its low median FGA, whereas the Chilean, Japanese, and South Korean cohorts exhibited more extensive alterations. The Indian cohort was highly heterogeneous, including both near-diploid and highly altered tumours. A complete summary of significant GISTIC2 focal lesions (q < 0.25) is provided in Supplementary Table S4. Across cohorts, recurrent focal events were more consistently observed for deletions than for amplifications, particularly at 19p13.3, which was found in the Indian, Japanese, and South Korean cohorts (Supplementary Table S5).

Nine tumours from the Indian cohort showed no gene mutations (see Fig. 2). Copy-number analysis revealed heterogeneous genomic profiles among these samples, with four tumours showing diploid profiles, four exhibiting an FGA > 0.10, and one showing an FGA of 0.02. Detailed copy-number profiles per-sample are provided in Supplementary Table S6.

### Geographic and genetic differences in GBC mutation profiles

We then assessed differences between cohorts in the frequency of recurrent gene mutations and mutational signatures. Three genes showed differences in their mutation frequencies: *ELF3*, *MACF1*, and *MUC16*. *ELF3* mutations were most frequent in South Korea (31%), *MACF1* mutations were most frequent in China (20%), and the frequency of *MUC16* mutations showed the strongest cohort differences (p = 0.007 [Wald Chi-Square test]), being most frequent in China (51%). The frequency of four mutational signatures (SBS5, SBS10b, SBS13, and SBS29) also varied across cohorts. Among these, SBS29 showed the strongest cohort heterogeneity (p < 0.00001 [Chi-Square test]), being most frequent in China (55%) and rare (2%) in India and South Korea (Table 4).

**Table 4:** Differences between cohorts in the frequency of somatic mutations in specific genes, and in the frequency of mutational signatures.

Gene or mutational signature	Cohort	Samples	n	%	95% CI	p value [Chi-Square test]
*ELF3*	Chile	23	2	9	0–20	0.04
	China	51	6	12	3–21	
	India	59	4	7	0–13	
	Japan	38	5	13	2–24	
	South Korea	91	28	31	21–40	
*MACF1*	Chile	23	1	4	0–13	0.04
	China	51	10	20	9–31	
	India	59	1	2	0–5	
	Japan	38	4	11	1–20	
	South Korea	91	9	10	4–16	
*MUC16*	Chile	23	2	9	0–20	0.007
	China	51	26	51	37–65	
	India	59	7	12	4–20	
	Japan	38	8	21	8–34	
	South Korea	91	14	15	8–23	
SBS5	Chile	23	15	65	46–85	5 × 10^−4^
	China	51	48	94	88–100	
	India	59	38	64	52–77	
	Japan	38	23	61	45–76	
	South Korea	91	68	75	66–84	
SBS10b	Chile	23	6	26	8–44	0.05
	China	51	1	2	0–6	
	India	59	5	8	1–16	
	Japan	38	2	5	0–12	
	South Korea	91	15	16	9–24	
SBS13	Chile	23	2	9	0–20	0.05
	China	51	10	20	9–31	
	India	59	27	46	33–58	
	Japan	38	16	42	26–58	
	South Korea	91	49	54	44–64	
SBS29	Chile	23	6	26	8–44	<0.0001
	China	51	28	55	41–69	
	India	59	1	2	0–5	
	Japan	38	10	26	12–40	
	South Korea	91	2	2	0–5	

Frequencies and their corresponding 95% confidence intervals (CI) are shown by cohort. p value, probability value from a multivariable logistic regression model that included study cohort, age, sex, gallstones, tumour stage, and coverage in tumour and normal tissues as explanatory variables. Only genes and mutational signatures with p value < 0.05 are shown. *ELF3*, E74 like ETS transcription factor 3 gene; *MACF1*, microtubule actin crosslinking factor 1 gene; *MUC16*, mucin 16, cell surface associated gene; SBS, single-base substitution.

After examining the differences in GBC mutation profiles between cohorts, we performed more detailed analyses to investigate possible association with the major subtypes of genetic ancestry in the investigated cohorts. Multiple linear regression analyses revealed that TMB increased by 0.08 (95%CI 0.03–0.14) for every 1% increase in the proportion of Japanese ancestry (p = 0.005 [F-test], Supplementary Fig. S20). According to multiple logistic regression, each 1% increase in the proportion of Japanese ancestry was associated with an increased risk of mutations in *MACF1* (by 2%) and *MUC16* (by 1%), with a decreased risk of SBS5 (by 2%), and with an increased risk of SBS29 (by 7%; p ≤ 0.0001 [Chi-Square test], Table 5). Every 1% increase in the proportion of Tujia-Chinese ancestry was associated with an increased risk of mutations in *MUC16* (by 1%) and SBS5 (by 3%). Each 1% increase in the proportion of Xibo-South Korean ancestry was associated with a 2% increased risk of SBS5, a 1% increased risk for the occurrence of the APOBEC signature SBS13, and a 2% decreased risk of SBS29. The frequencies of potentially actionable mutations in the investigated cohorts of gallbladder cancer patients are shown in Table 6.

**Table 5:** Association between the individual proportions of ancestry subtypes and the frequency of somatic mutations in specific genes, as well as between ancestry proportions and the frequency of mutational signatures.

Gene or mutational signature	n	%	Ancestry subtype	OR^a^	95% CI	p value [Chi-Square test]
*MACF1*	25	10	Japanese	**1.02**	1.00–1.04	0.02
			Lahu	1.02	0.99–1.04	0.23
*MUC16*	57	22	Japanese	**1.01**	1.00–1.03	0.04
			Lahu	1.01	0.99–1.03	0.38
			She	1.00	0.98–1.02	0.99
			Tujia	**1.01**	1.00–1.03	0.04
SBS5	192	73	Lahu	0.85	0.67–1.09	0.20
			Japanese	**0.98**	0.97–0.99	0.02
			She	1.00	0.96–1.03	0.81
			Tujia	**1.03**	1.01–1.05	0.01
			Xibo	**1.02**	1.01–1.03	0.004
SBS13	104	40	Japanese	0.99	0.97–1.00	0.09
			Xibo	**1.01**	1.00–1.03	0.04
SBS29	47	18	Japanese	**1.07**	1.04–1.10	<0.0001
			Xibo	**0.98**	0.97–0.99	0.03

a ORs represent the change in the risk of carrying a mutation or mutational signature per 1% increase in the proportion of ancestry. They were estimated using a multiple logistic regression model that included the individual proportion of ancestry, age, sex, gallstones, tumour stage, and coverage in tumour and normal tissues as explanatory variables. Results are shown only for the major ancestry subtypes in the Chinese and South Korean cohorts, as no additional associations were observed at the 5% level of statistical significance. Bold type denotes associations for which the 95% confidence interval (CI) did not include 1.00. OR, odds ratio; *MACF1*, microtubule actin crosslinking factor 1 gene; *MUC16*, mucin 16, cell surface associated gene; SBS, single-base substitution.

**Table 6:** Frequencies of potentially actionable mutations in the investigated cohorts of gallbladder cancer patients.

Gene	Chile (n = 23)	China (n = 51)	India (n = 59)	Japan (n = 38)	South Korea (n = 91)	Examples of biomarker-informed therapeutic options
*TP53*	9 (39%)	15 (29%)	**16 (27%)**	15 (39%)	**42 (46%)**	atorvastatin, arsenic trioxide, vorinostat, adavosertib, lamivudine
*MUC16*	**2 (9%)**	**26 (51%)**	7 (12%)	8 (21%)	14 (15%)	oregovomab, DMUC5754A
*ERBB2*	**4 (17%)**	3 (6%)	5 (8%)	**2 (5%)**	14 (15%)	trastuzumab, neratinib, pertuzumab plus trastuzumab, zanidatamab
*ARID1A*	**4 (17%)**	5 (10%)	**5 (8%)**	**3 (8%)**	9 (10%)	durvalumab
*ERBB3*	**1 (4%)**	3 (6%)	5 (8%)	3 (8%)	**8 (9%)**	patritumab, seribantumab, lumretuzumab
*BRCA2*	**0 (0%)**	**5 (10%)**	4 (7%)	1 (3%)	6 (7%)	PARP inhibitors: olaparib, talazoparib, rucaparib, niraparib
*PIK3CA*	1 (4%)	**4 (8%)**	**5 (8%)**	2 (5%)	**3 (3%)**	alpelisib, capivasertib
*NF1*	**3 (13%)**	3 (6%)	2 (3%)	**0 (0%)**	5 (5%)	selumetinib
*KRAS*	**0 (0%)**	2 (4%)	**4 (7%)**	**0 (0%)**	2 (2%)	KRAS G12C: sotorasib, adagrasib
*BRCA1*	**0 (0%)**	**2 (4%)**	1 (2%)	1 (3%)	1 (1%)	PARP inhibitors: olaparib, talazoparib, rucaparib, niraparib
*BRAF*	**0 (0%)**	**0 (0%)**	**1 (2%)**	**0 (0%)**	1 (1%)	BRAF V600E: dabrafenib plus trametinib

Bold type indicates the cohorts with the lowest and highest mutation frequencies. *TP53*, tumour protein p53 gene; *MUC16*, mucin 16, cell surface associated gene; *ERBB2*, erb-b2 receptor tyrosine kinase 2 gene; *ARID1A*, AT-rich interaction domain 1A gene; *ERBB3*, erb-b2 receptor tyrosine kinase 3 gene; *BRCA2*, BRCA2 DNA repair associated gene; *PIK3CA*, phosphatidylinositol-4,5-bisphosphate 3-kinase catalytic subunit alpha gene; *NF1*, neurofibromin 1 gene; *KRAS*, Kirsten rat sarcoma viral oncogene homologue gene; *BRCA1*, BRCA1 DNA repair associated gene; *BRAF*, B-Raf proto-oncogene serine/threonine kinase gene.

Among 262 cases, 249 (95%) tumour samples were fresh frozen and 13 (5%) were FFPE, whereas 190 (72.5%) matched normal samples were adjacent normal tissue and 72 (27.5%) were blood. We found no association between the frequency of specific somatic mutations or mutational signatures, and the type of tumour sample (fresh-frozen vs. FFPE) or the type of normal sample (adjacent normal vs. tissue blood) (p > 0.25 [Chi-Square test]). However, the TMB in fresh-frozen samples (median 2.5) was higher than in FFPE tumour samples (median 1.28, p = 0.0002 [F-test]), and the FGA was lower in fresh-frozen samples (median 0.2) than in FFPE tumour samples (median 0.6, p = 0.003 [F-test]) (Supplementary Table S7).

## Discussion

GBC is a very aggressive malignancy. Despite its low prevalence globally, GBC represents a substantial public health burden in certain low-income and middle-income regions from South America, and South and East Asia, emphasising the need for a deeper understanding of the environmental and genetic factors contributing to the development and heterogeneity of this disease. In the present analysis of worldwide whole-exome sequencing data, we examined geographic and genetic differences in TMB, FGA, and the frequency of recurrent mutations and mutational signatures, resulting from specific mutagenesis processes and cancer risk factors, in patient cohorts from Chile, China, India, Japan and South Korea.

ADMIXTURE analyses identified nine subpopulations from 1000G and HGDP as the best surrogates for genetic ancestry in the investigated cohorts. Previous studies have found a strong association between GBC risk and the individual proportion of Indigenous American ancestry, particularly Mapuche (the Mapuche are the largest group of Indigenous people in Chile).^5^^,^^18^ However, in order to facilitate the reproducibility of our results using publicly available data, and given that the study included only 23 Chilean samples, we incorporated Peruvian individuals from 1000 GP with an estimated 100% Indigenous American ancestry into the reference panel for ancestry estimation. The major subtypes of genetic ancestry in the Indian cohort were represented by the Punjabi and Bengali subpopulations, located predominantly in the northern Indo-Gangetic Belt of India. Previous epidemiological studies have examined the high incidence of GBC in these North Indian communities, suggesting that factors related to their environmental exposure and ancestral background may contribute to the increased GBC prevalence in this area.^32^^,^^33^ Interestingly, the major ancestry subtype in the Japanese cohort of GBC patients was the Lahu, an Indigenous people originating from the Yunnan region of southwest China. The genetic makeup of the South Korean cohort reflects its complex ancestry, which included contributions from the Xibo subpopulation in China and the Japanese subpopulation in Japan. This multifaceted ancestral background may help explain the unique mutation patterns observed in South Korean GBC patients.^34^

The mutation profiles identified in this worldwide GBC exome study are consistent with previously published results.^13^^,^^35^ For example, in line with previous findings, the prevalence of *ELF3* mutations was highest in the East Asian GBC patients. A central aspect of the present study was the investigation of geographic and genetic heterogeneity in the mutation profiles: the South Korean cohort exhibited the highest frequency of *ELF3* mutations (31%), compared with 12–13% in the Chinese and Japanese cohorts. Another example of mutation heterogeneity was *MUC16* mutations, which were found in 51% of patients in the Chinese cohort, compared with just 9% of Chilean patients.^36^ On a finer scale, we observed an association between the patient’s proportion of Tujia ancestry and the frequency of mutations in the *MUC16* gene. Interestingly, *MUC16* mutations have previously been described as relevant to other types of cancer in the Chinese population.^37^

The frequency of common mutational signatures observed in this study is also consistent with the mutation mechanisms previously proposed for GBC,^12^^,^^13^^,^^38^ but this study allowed geographic and genetic differences to be assessed. For instance, mutational signatures associated with APOBEC-mediated cytidine deamination were present in the five cohorts studied, but showed differences in frequency: SBS13 was found in 54% of South Korean versus 9% of Chilean GBC tumours, possibly reflecting distinct aetiologies across the different cohorts. A more nuanced finding was the observed correlation between an increasing proportion of Xibo ancestry and an increased SBS13 frequency. Conversely, the higher the proportion of Japanese ancestry, the lower the frequency of the ageing-related SBS5 signature. The geographic and genetic differences in mutational signatures identified in this study emphasise the importance of considering understudied Asian minorities in future GBC research. The Tujia, for example, are the eighth-largest ethnic minority in China; they are mainly located in the western Hunan and southwestern Hubei provinces, and are genetically related to the She, another minority group distributed in southern China.^39^

Focusing on the subset of gene mutations investigated, we briefly highlight potential geographic differences in treatment options. For example, GBC patients with a high proportion of Tujia-Chinese ancestry showed the highest frequency of *MUC16* mutations and would particularly benefit from treatment with oregovomab and DMUC5754A.^40^ Patients with tumours harbouring *ERBB2* mutations benefit from targeted treatment with trastuzumab, neratinib and the combination of pertuzumab and trastuzumab, and 17% of Chilean and 15% of South Korean GBC tumours exhibited an *ERBB2* mutation^41^, ^42^, ^43^, ^44^, ^45^ (Table 6). The Chilean cohort displayed the highest frequency of *ARID1A* and *NF1* mutations among the populations studied, and targeted treatment with durvalumab or selumetinib, respectively, could be particularly relevant for Chilean GBC patients.^46^^,^^47^ The Chinese and Indian cohorts showed the highest frequency of *PIK3CA* mutations, highlighting the potential of alpelisib and capivasertib treatments in China and India.^48^ Given the frequency of *BRCA2* mutations in China, Chinese GBC patients may particularly benefit from treatment with PARP inhibitors, including olaparib, talazoparib, rucaparib and niraparib.^49^^,^^50^ Tumour sequencing and subsequent targeted treatment strategies, tailored to regional mutation profiles, may help improve outcomes for GBC patients from ethnic minorities, particularly in resource-poor areas.^51^

The variation observed in clinically relevant alterations between cohorts suggests that the actionable landscape of GBC varies across different populations. We compared our findings with publicly available data from the Gallbladder Carcinoma MSK cohort, although this dataset was generated using the MSK-IMPACT targeted sequencing platform and included both primary and metastatic tumours. Taking these limitations into account, the frequency of *ARID1A* mutations in the MSK cohort (18%) was most similar to the frequency observed in the Chilean cohort (17%). The frequency of *PIK3CA* mutations in MSK (9%) most closely resembled the Chinese and Indian frequencies (8% each), and the frequency of *KRAS* mutations in the MSK cohort (7%) matched the Indian frequency (7%). If validated in future studies, the identified differences in frequency of actionably mutations could serve as a basis for the design of targeted treatment strategies at diverse populations, such as that in the US.

A key strength of this study was the use of a unified pipeline to analyse whole-exome sequencing data from the five cohorts. By restricting the analyses to the exome region common to all cohorts and selecting somatic mutations identified by both Mutect2 and VarScan2, we aimed to improve comparability between datasets generated under distinct conditions. Most precision oncology research has focused on patients of European descent, leading to growing inequalities in the application of targeted therapies for patients with different genetic backgrounds. This study addressed genetic diversity in genomic studies so that precision oncology can be adapted to underrepresented populations.^51^ The study also had several limitations. Despite harmonisation, the cohorts differed in terms of sample size, the type of tumour and normal tissue samples, and sequencing methodology. Restriction to the shared exome region was intended to improve comparability, but excluded cohort-specific callable regions and may have reduced mutation counts. The main components of genetic ancestry in the Chinese cohort (Tujia, She and Lahu) accounted for only 42% of genetic variability; however, for the sake of simplicity, we did not consider subpopulations contributing less than 10% to the ancestry (e.g., 9% average proportion of Xibo ancestry in the Chinese cohort). Some ancestry analyses were based on relatively small subgroups. Finally, whole-exome sequencing does not capture all genomic mechanisms relevant to GBC, including non-coding, transcriptomic, and epigenetic alterations.

In conclusion, GBC, which occurs predominantly in low-income and middle-income regions of Asia and Latin America, shows considerable geographic and genetic diversity in mutation profiles. We observed cohort variability in TMB, FGA, frequently mutated genes such as *MUC16*, and in mutational signatures such as SBS5 and SBS29. These differences were associated with specific genetic ancestries; for example, Tujia-Chinese ancestry correlated with the frequency of *MUC16* mutations, while Japanese ancestry was associated with the frequency of SBS5. The study emphasises the urgent need for more diverse and inclusive genomic studies to develop effective precision oncology strategies for ethnically underrepresented patients.

## Contributors

Valentina Gárate-Calderón: Conceptualisation, investigation, formal analysis, methodology, access to and verification of the underlying data, writing-original draft, and funding acquisition. Rajiv Kumar: Resources and writing-review & editing. Katherine Marcelain: Resources and writing-review & editing. Linda Zollner: Formal analysis, resources, and writing-review & editing. Felix Boekstegers: Resources and writing-review & editing. Olga Barajas: Resources and writing-review & editing. Denisse Loader: Resources and writing-review & editing. María Teresa Rivera: Resources and writing-review & editing. Erik Morales: Resources and writing-review & editing. Gonzalo de Toro: Resources and writing-review & editing. Christian Caglevic: Conceptualisation, resources, and writing-review & editing. Tatsuhiro Shibata: Resources and writing-review & editing. Justo Lorenzo Bermejo: Conceptualisation, supervision, methodology, resources, access to and verification of the underlying data, writing-review & editing, and funding acquisition. Valentina Gárate-Calderón and Justo Lorenzo Bermejo directly accessed and verified the underlying data reported in the manuscript. All authors had full access to all the data in the study, read and approved the final version of the manuscript, and accept responsibility for the decision to submit for publication.

## Data sharing statement

De-identified genomic data from previously published cohorts are available through the original controlled-access repositories. Data from the Shanghai/Chile cohort^12^ are available through dbGaP under accession phs001404.v1.p1. Data from the Korean, Indian, and Chilean cohort reported by Pandey and colleagues^13^ are available through the European Genome-phenome Archive under accession EGAS00001003004. Japanese cohort data reported by Nakamura and colleagues^9^ are available through the European Genome-phenome Archive under accession EGA00001000950 and through the International Cancer Genome Consortium database, and additional Japanese gallbladder cancer data reported by Wardell and colleagues^11^ are available through the Japanese Genotype-phenotype Archive under submission JGA00000000119 (study JGAS00000000109; datasets JGAD00000000117-JGAD00000000118). The 13 additional Chilean tumour–normal pairs generated by the Statistical Genetics Research Group at Heidelberg University are available upon reasonable request from the corresponding author, subject to ethics approval and data-sharing agreements.

## Declaration of interests

The authors declare no competing interests.
