# Supplementary material for: Geographic and genetic diversity in gallbladder cancer mutation profiles: insights from a worldwide exome analysis
Source: eBioMedicine. 2026 May 22;128:106305. doi: 10.1016/j.ebiom.2026.106305 (PMC13199658; doi:10.1016/j.ebiom.2026.106305)
Supplement: Supplementary Figs. S1–S20 and Tables S1–S7 [file mmc1.pdf]

Supplementary material

Table of Contents

Investigated GBC cohorts 3

Supplementary Table S1. Exome capture kits and mean target coverage across cohorts, showing values reported in the original publications and values recalculated within the harmonised shared region. 4

Supplementary Table S2. Cosine similarity across gallbladder cancer cohorts 5

Supplementary Table S3. Sample-level mutational signature assignment metrics across the study cohort 6

Supplementary Table S4. Cohort-level summary of significant focal copy-number alteration peaks identified by GISTIC2. 15

Supplementary Table S5. Recurrent significant focal copy-number alteration peaks identified by GISTIC2 across cohorts. 16

Supplementary Table S6. Copy-number profiles of tumours without detectable nonsynonymous somatic SNVs/indels. 17

Supplementary Table S7. Distribution of tumour mutation burden (TMB) and the fraction of genome altered (FGA) according to tumour sample type. 18

Supplementary Figure S1. Geographic location of the gallbladder cancer cohorts and the 1000 Genomes/HGDP reference populations used for ancestry inference 19

Supplementary Figure S2. Genetic principal component analysis of the five investigated cohorts of gallbladder cancer patients and the populations of the 1000 Genomes Project and the Human Genome Diversity Project. 20

Supplementary Figure S3· Supervised ADMIXTURE ancestry estimates for Chilean gallbladder cancer patients using 1000 Genomes reference panels. 21

Supplementary Figure S4· Model selection and major ancestry subtypes in the Chilean cohort. 22

Supplementary Figure S5· Model selection and major ancestry subtypes in the Chinese cohort. 23

Supplementary Figure S6· Model selection and major ancestry subtypes in the Indian cohort. 24

Supplementary Figure S7· Model selection and major ancestry subtypes in the Japanese cohort. 25

Supplementary Figure S8· Model selection and major ancestry subtypes in the South Korean cohort. 26

Supplementary Figure S9· Principal component analysis of Asian gallbladder cancer cohorts and HGDP reference panels for ancestry inference. 27

Supplementary Figure S10· Mutational signatures identified in the Chilean cohort. 28

Supplementary Figure S11· Mutational signatures identified in the Chinese cohort 29

Supplementary Figure S12· Mutational signatures identified in the Indian cohort 30

Supplementary Figure S13· Mutational signatures identified in the Japanese cohort 31

Supplementary Figure S14· Mutational signatures identified in the South Korean cohort. 32

Supplementary Figure S15. Cohort-level copy-number profiles and GISTIC2 analysis in the Chilean cohort. 33

Supplementary Figure S16. Cohort-level copy-number profiles and GISTIC2 analysis in the Chinese cohort. 34

Supplementary Figure S17. Cohort-level copy-number profiles and GISTIC2 analysis in the Indian cohort. 35

Supplementary Figure S18. Cohort-level copy-number profiles and GISTIC2 analysis in the Japanese cohort. 36

Supplementary Figure S19. Cohort-level copy-number profiles and GISTIC2 analysis in the South Korean cohort. 37

Supplementary Figure S20. Association between the proportion of Japanese ancestry and tumour mutational burden (TMB). **38**

References **39**

Investigated GBC cohorts

We obtained access to whole-exome sequencing data from 249 of the 262 paired GBC tumour–normal samples analysed in this study from published studies [1–4] (**Supplementary Table S1**). The Japanese sample HK161[2] was excluded from the analysis because, despite using the same pipeline to call somatic variants, all detected variants were filtered out due to low quality, resulting in no somatic variants being retained. Our genetic analyses revealed that four patients designated as Chinese in the Nepal *et al.* [4] study were Chilean, and four patients designated as Chilean were Chinese.

Our analysis of somatic variants revealed some minor discrepancies with the mutation frequencies reported in the original studies. The reason for these discrepancies could be due to the stringent filters we applied in our unified analysis pipeline. For example, we considered a sequencing depth of more than 30× for somatic mutations. This sequencing depth threshold may have excluded some low depth mutations, potentially leading to an underestimation of the frequency of somatic mutations in our analysis, but we have confidence in the variants we identified. The use of different bioinformatics tools and variant calling parameters in the original studies and our unified pipeline could also account for some differences in mutation profiles. For instance, our results for the 10 Japanese samples from Wardell *et al.*[2] are more similar to the results of a more recent publication in which these samples were analysed according to GATK best practices [5], in contrast to the analytical approach used in the original study.

Supplementary Table S1. Exome capture kits and mean target coverage across cohorts, showing values reported in the original publications and values recalculated within the harmonised shared region.

Cohort	n	Exome capture kit	Reported depth (Normal/Tumour)	Shared region depth (Normal/Tumour)	Source / notes
Chile	23	SureSelect XT Human All Exon V6 (Agilent Technologies)	100/200x	130/176x	13 newly sequenced Chilean samples from our Statistical Genetic Group. Heidelberg
Chile	23	SureSelect XT Human All Exon V5 (Agilent Technologies)	100/100x	105/104x	2 samples from Pandey, *et al.* 2020
Chile	23	Nimblegen SeqCap EZ Exome + UTR (Roche)	150/150x	44/98x	8 samples from Nepal, *et al*. 2021
China	51	Nimblegen SeqCap EZ Exome + UTR (Roche)	150/150x	44/98x	51 samples from Nepal, *et al.* 2021
India	59	SureSelect XT Human All Exon V5 (Agilent Technologies)	100/100x	105/104x	59 samples from Pandey, *et al.* 2020
Japan	38	SureSelect XT Human All Exon V5 (Agilent Technologies)	100/100x	106/108x	28 samples from Nakamura, *et al.* 2015
Japan	38	Nextera Rapid Capture Exomes (Illumina).	50/100x	81/100x	10 samples from Wardell, *et al.* 2018
South Korea	91	SureSelect XT Human All Exon V5 (Agilent Technologies)	100/100x	105/104x	91 Samples from Pandey, *et al.* 2020

Note: “Reported depth” corresponds to the sequencing depth stated in each original publication (mean/median target coverage or intended depth, as reported). “Shared region depth” was recalculated from the aligned BAM files within the shared region (32.12 Mb) using a consistent coverage metric across cohorts.

Supplementary Table S2. Cosine similarity across gallbladder cancer cohorts

Cohort	Samples, n	Median cosine similarity	Minimum cosine similarity	Maximum cosine similarity
Chile	23	0·827	0·61	0·95
China	51	0·851	0·71	0·99
India	52	0·759	0·25	0·96
Japan	38	0·879	0·54	0·98
South Korea	91	0·783	0·33	0·98

n: number of samples. The number of Indian samples included in signature assignment analyses may differ from the total cohort size because only tumours with sufficient filtered Mutect2 SNVs were evaluable for sample-level signature reconstruction.

Supplementary Table S3. Sample-level mutational signature assignment metrics across the study cohort

Sample_ID	Cohort	Total mutations used for assignment	Sample cosine similarity	Assigned COSMIC SBS signatures
GBC012	Chile	25	0·781	SBS42
GBC021	Chile	50	0·888	SBS2; SBS5; SBS13; SBS39
GBC_0260	Chile	47	0·841	SBS29
GBC_0296	Chile	119	0·88	SBS5; SBS29
GBC_0315	Chile	126	0·907	SBS5; SBS18; SBS59
GBC_0333	Chile	34	0·814	SBS5; SBS18; SBS52
GBC_A000156	Chile	110	0·84	SBS1; SBS3; SBS5; SBS29
GBC_A000185	Chile	52	0·826	SBS10a; SBS24; SBS50
GBC_A000186	Chile	158	0·949	SBS1; SBS4; SBS5; SBS29; SBS48; SBS59
GBC_A000191	Chile	41	0·663	SBS5; SBS29
HEI0021	Chile	102	0·865	SBS1; SBS7c; SBS30; SBS85
HEI0022	Chile	73	0·81	SBS1; SBS5; SBS10b; SBS15; SBS29
HEI0032	Chile	13	0·775	SBS1; SBS6; SBS10b
HEI0035	Chile	74	0·827	SBS1; SBS5; SBS15; SBS39; SBS50
HEI0036	Chile	86	0·774	SBS7a; SBS15; SBS39
HEI0037	Chile	50	0·724	SBS1; SBS2; SBS5; SBS10a; SBS13; SBS32
HEI0043	Chile	65	0·771	SBS1; SBS5; SBS15
HEI0044	Chile	186	0·94	SBS1; SBS2; SBS5; SBS6; SBS7b; SBS10b
HEI0046	Chile	80	0·846	SBS5; SBS10b; SBS15; SBS18
HEI0048	Chile	43	0·754	SBS6; SBS10b; SBS39
HEI0123	Chile	20	0·612	SBS5; SBS6
HEI0125	Chile	64	0·878	SBS1; SBS5; SBS10b; SBS42
GBC_0369	China	119	0·933	SBS5; SBS7a; SBS7c; SBS29; SBS52
GBC_0434	China	150	0·883	SBS1; SBS5; SBS29; SBS42
GBC_0448	China	152	0·845	SBS5; SBS18; SBS35; SBS50
GBC_0506	China	77	0·839	SBS5; SBS29; SBS58
GBC_0551	China	112	0·883	SBS1; SBS5; SBS18; SBS24; SBS31
GBC_0559	China	89	0·714	SBS1; SBS5; SBS7a

GBC_0584	China	102	0·821	SBS1; SBS5; SBS7a; SBS24; SBS54
GBC_0687	China	112	0·816	SBS1; SBS5; SBS18; SBS31
GBC_0701	China	186	0·883	SBS1; SBS5; SBS7b; SBS13; SBS29; SBS46
GBC_0820	China	104	0·854	SBS5; SBS29; SBS30
GBC_0824	China	83	0·836	SBS5; SBS6; SBS29
GBC_0826	China	111	0·886	SBS1; SBS5; SBS24; SBS36
GBC_0834	China	94	0·759	SBS5; SBS29
GBC_0859	China	104	0·764	SBS1; SBS5; SBS18; SBS50
GBC_0860	China	83	0·81	SBS1; SBS5; SBS29
GBC_0899	China	99	0·805	SBS1; SBS5; SBS7a; SBS24
GBC_0973	China	59	0·709	SBS5; SBS18
GBC_0982	China	83	0·836	SBS1; SBS5; SBS29; SBS54
GBC_1033	China	106	0·836	SBS1; SBS2; SBS5; SBS13; SBS24
GBC_1040	China	62	0·872	SBS1; SBS5; SBS29; SBS54; SBS59; SBS60
GBC_1070	China	137	0·851	SBS5; SBS29; SBS50
GBC_1137	China	77	0·749	SBS5; SBS23; SBS29; SBS46
GBC_1166	China	137	0·883	SBS1; SBS2; SBS4; SBS5; SBS10b; SBS13; SBS59; SBS84
GBC_1172	China	75	0·788	SBS1; SBS5; SBS24; SBS54
GBC_1173	China	111	0·847	SBS1; SBS5; SBS24; SBS48; SBS50
GBC_1192	China	124	0·851	SBS5; SBS11; SBS29
GBC_1223	China	158	0·857	SBS5; SBS29; SBS44
GBC_1303	China	111	0·901	SBS5; SBS6; SBS13; SBS18; SBS50; SBS59
GBC_1341	China	95	0·858	SBS29; SBS32; SBS39; SBS54; SBS59
GBC_1404	China	206	0·964	SBS2; SBS5; SBS13; SBS29
GBC_1410	China	67	0·848	SBS4; SBS5; SBS54; SBS59
GBC_1456	China	206	0·91	SBS2; SBS5; SBS13; SBS29
GBC_1483	China	83	0·874	SBS5; SBS29; SBS37
GBC_1499	China	101	0·863	SBS5; SBS7a; SBS29
GBC_1548	China	91	0·862	SBS6; SBS7b; SBS7d; SBS39; SBS54
GBC_1609	China	94	0·896	SBS1; SBS5; SBS7a; SBS7c; SBS13; SBS29
GBC_1648	China	138	0·909	SBS5; SBS29

GBC_1740	China	83	0·848	SBS5; SBS29; SBS30
GBC_1785	China	65	0·843	SBS1; SBS10a; SBS13; SBS29; SBS42
GBC_1829	China	123	0·894	SBS5; SBS29
GBC_1862	China	59	0·772	SBS1; SBS5; SBS18
GBC_1883	China	125	0·871	SBS1; SBS4; SBS5; SBS59
GBC_1916	China	892	0·988	SBS1; SBS2; SBS5; SBS13
GBC_1975	China	138	0·865	SBS1; SBS5; SBS29; SBS39
GBC_3050	China	128	0·903	SBS5; SBS24; SBS50
GBC_3083	China	86	0·796	SBS1; SBS5; SBS11; SBS29; SBS54
GBC_3120	China	61	0·779	SBS1; SBS2; SBS5; SBS13; SBS21; SBS31; SBS39
GBC_A000024	China	59	0·748	SBS5; SBS24; SBS50
GBC_A000033	China	84	0·799	SBS2; SBS5; SBS35; SBS59
GBC_A000034	China	132	0·918	SBS1; SBS5; SBS29; SBS36
GBC_A000076	China	65	0·787	SBS5; SBS29; SBS32
GBC001	India	74	0·752	SBS1; SBS7a; SBS13; SBS39
GBC002	India	134	0·849	SBS2; SBS5; SBS13; SBS29; SBS39
GBC003	India	128	0·914	SBS1; SBS2; SBS5; SBS13; SBS17b
GBC004	India	58	0·811	SBS5; SBS7a; SBS7c; SBS16
GBC005	India	53	0·82	SBS2; SBS5; SBS13; SBS15
GBC006	India	42	0·861	SBS1; SBS2; SBS5; SBS13; SBS39
GBC007	India	534	0·947	SBS1; SBS5; SBS15; SBS42
GBC008	India	19	0·559	SBS5; SBS15; SBS18
GBC009	India	44	0·677	SBS1; SBS2; SBS5; SBS13; SBS39
GBC011	India	49	0·853	SBS1; SBS2; SBS5; SBS7a; SBS13
GBC013	India	29	0·717	SBS5; SBS15; SBS17b; SBS31
GBC014	India	49	0·875	SBS1; SBS2; SBS5; SBS13; SBS15
GBC016	India	10	0·553	SBS51
GBC017	India	46	0·745	SBS1; SBS2; SBS5; SBS13; SBS39
GBC020	India	117	0·749	SBS17b; SBS25; SBS27
GBC030	India	56	0·833	SBS1; SBS2; SBS5; SBS13; SBS42
GBC031	India	68	0·937	SBS1; SBS2; SBS5; SBS13

GBC032	India	20	0·532	SBS1; SBS5; SBS42
GBC034	India	1	0·685	SBS42
GBC035	India	130	0·931	SBS1; SBS2; SBS5; SBS13
GBC036	India	13	0·516	SBS5; SBS42
GBC038	India	1	0·247	SBS42
GBC039	India	48	0·811	SBS1; SBS2; SBS39
GBC040	India	1	0·275	SBS37
GBC042	India	29	0·725	SBS2; SBS14; SBS39
GBC043	India	71	0·905	SBS2; SBS5; SBS13; SBS17b; SBS42
GBC044	India	34	0·855	SBS4; SBS10b; SBS15; SBS49
GBC047	India	35	0·801	SBS1; SBS2; SBS5; SBS10a; SBS10b; SBS13
GBC048	India	42	0·713	SBS1; SBS2; SBS5; SBS13; SBS14
GBC049	India	61	0·769	SBS5; SBS7a; SBS13
GBC050	India	68	0·917	SBS2; SBS6; SBS10a; SBS10b; SBS13; SBS39
GBC052	India	22	0·666	SBS1; SBS2; SBS5; SBS13; SBS50
GBC054	India	61	0·839	SBS1; SBS2; SBS5; SBS13; SBS15
GBC171	India	55	0·766	SBS1; SBS13; SBS42
GBC183	India	25	0·597	SBS1; SBS5; SBS13; SBS28
GBC187	India	7	0·518	SBS5; SBS10b; SBS54
GBC192	India	42	0·705	SBS2; SBS5; SBS13; SBS25
GBC194	India	52	0·96	SBS20; SBS45
GBC196	India	103	0·707	SBS42; SBS54
GBC197	India	63	0·859	SBS1; SBS2; SBS5; SBS6; SBS7b; SBS13; SBS17b
GBC200	India	14	0·485	SBS5; SBS39
GBC201	India	32	0·64	SBS1; SBS5
GBC203	India	49	0·672	SBS1; SBS5; SBS13; SBS30
GBC208	India	113	0·914	SBS1; SBS2; SBS5; SBS7a; SBS13; SBS17b
GBC212	India	46	0·848	SBS1; SBS5; SBS15; SBS17b
GBC213	India	22	0·571	SBS2; SBS5; SBS24
GBC214	India	7	0·769	SBS5; SBS10a; SBS59
GBC215	India	79	0·867	SBS1; SBS10b; SBS13; SBS50

GBC216	India	56	0·901	SBS1; SBS2; SBS5; SBS13; SBS17b; SBS42
GBC217	India	9	0·52	SBS1; SBS5; SBS50; SBS59
GBC218	India	46	0·685	SBS5; SBS6; SBS14; SBS39
GBC219	India	12	0·592	SBS5; SBS15; SBS49
BD170	Japan	33	0·793	SBS1; SBS2; SBS5; SBS13
BD171	Japan	66	0·914	SBS1; SBS2; SBS5; SBS13; SBS15
BD172	Japan	41	0·888	SBS1; SBS2; SBS5; SBS13
BD173	Japan	268	0·827	SBS7c; SBS20; SBS24; SBS57
BD174	Japan	52	0·902	SBS1; SBS2; SBS5; SBS7a; SBS13
BD175	Japan	113	0·916	SBS1; SBS2; SBS5; SBS13
BD176	Japan	45	0·785	SBS5; SBS13; SBS15
BD177	Japan	97	0·915	SBS1; SBS2; SBS5; SBS13
BD178	Japan	69	0·955	SBS2; SBS5; SBS6; SBS13
BD179	Japan	136	0·772	SBS1; SBS5; SBS39; SBS42
BD180	Japan	145	0·638	SBS1; SBS5; SBS24; SBS39
BD181	Japan	11	0·633	SBS5; SBS6
BD182	Japan	649	0·965	SBS17b; SBS28; SBS46
BD183	Japan	126	0·882	SBS1; SBS2; SBS5; SBS13; SBS42; SBS50
BD184	Japan	29	0·725	SBS5; SBS13; SBS26; SBS38
BD185	Japan	29	0·8	SBS2; SBS5; SBS13; SBS15
BD186	Japan	39	0·78	SBS2; SBS5; SBS30
BD187	Japan	87	0·962	SBS1; SBS2; SBS5; SBS10b; SBS13
BD188	Japan	37	0·742	SBS1; SBS5; SBS10b; SBS15
BD189	Japan	121	0·841	SBS7a; SBS7c; SBS17b; SBS33; SBS39
BD190	Japan	48	0·889	SBS2; SBS5; SBS13; SBS20; SBS52
BD191	Japan	43	0·706	SBS1; SBS5; SBS17b; SBS39; SBS42
BD192	Japan	19	0·538	SBS1; SBS5; SBS39
BD193	Japan	4	0·777	SBS10a; SBS15
BD194	Japan	26	0·805	SBS1; SBS2; SBS13; SBS42
BD202	Japan	40	0·711	SBS1; SBS7a; SBS7b; SBS42
BD206	Japan	25	0·859	SBS1; SBS2; SBS5; SBS13

BD246	Japan	193	0·98	SBS1; SBS2; SBS5; SBS7a; SBS13
HK74	Japan	270	0·96	SBS29
HK83	Japan	118	0·931	SBS29; SBS50
HK85	Japan	212	0·915	SBS29
HK86	Japan	91	0·856	SBS29
HK87	Japan	62	0·875	SBS5; SBS29
HK88	Japan	50	0·908	SBS29
HK91	Japan	207	0·911	SBS29
HK92	Japan	66	0·923	SBS29; SBS59
HK93	Japan	300	0·929	SBS29
HK95	Japan	413	0·952	SBS29
GBC058	South Korea	32	0·82	SBS2; SBS5; SBS10a; SBS10b; SBS15; SBS52
GBC059	South Korea	165	0·639	SBS1; SBS5; SBS49
GBC060	South Korea	19	0·688	SBS5; SBS15
GBC061	South Korea	34	0·683	SBS1; SBS5; SBS18
GBC062	South Korea	23	0·74	SBS1; SBS2; SBS5; SBS13; SBS17a
GBC064	South Korea	60	0·709	SBS1; SBS5; SBS22; SBS58
GBC065	South Korea	2375	0·982	SBS1; SBS2; SBS5; SBS13
GBC066	South Korea	28	0·699	SBS1; SBS5; SBS7c; SBS13
GBC067	South Korea	27	0·776	SBS1; SBS23; SBS50; SBS52; SBS54
GBC068	South Korea	8	0·566	SBS42
GBC069	South Korea	27	0·833	SBS1; SBS2; SBS5; SBS13
GBC070	South Korea	30	0·699	SBS5; SBS6; SBS17b; SBS52
GBC071	South Korea	253	0·981	SBS1; SBS2; SBS5; SBS10b; SBS13
GBC072	South Korea	120	0·949	SBS1; SBS5; SBS17a; SBS17b
GBC073	South Korea	112	0·941	SBS2; SBS5; SBS7a; SBS13
GBC074	South Korea	281	0·982	SBS1; SBS2; SBS5; SBS10b; SBS13
GBC075	South Korea	29	0·712	SBS1; SBS2; SBS35
GBC076	South Korea	33	0·604	SBS5; SBS10b; SBS35
GBC077	South Korea	25	0·651	SBS1; SBS5; SBS13; SBS24
GBC078	South Korea	37	0·799	SBS5; SBS10b; SBS30

GBC080	South Korea	17	0·787	SBS1; SBS13; SBS15; SBS16; SBS50
GBC081	South Korea	30	0·569	SBS1; SBS5; SBS17b; SBS42
GBC082	South Korea	116	0·938	SBS1; SBS2; SBS5; SBS13
GBC083	South Korea	55	0·735	SBS5; SBS6; SBS13; SBS21; SBS50
GBC084	South Korea	174	0·901	SBS7a; SBS15; SBS36; SBS39
GBC085	South Korea	8	0·331	SBS1; SBS5; SBS57
GBC086	South Korea	43	0·786	SBS1; SBS7a; SBS13; SBS15
GBC087	South Korea	18	0·642	SBS1; SBS10b; SBS13
GBC088	South Korea	17	0·458	SBS5; SBS19
GBC089	South Korea	41	0·881	SBS1; SBS2; SBS5; SBS7a; SBS13
GBC090	South Korea	26	0·701	SBS1; SBS2; SBS5; SBS13
GBC091	South Korea	37	0·826	SBS1; SBS2; SBS5; SBS10b; SBS13
GBC092	South Korea	32	0·742	SBS1; SBS5; SBS10b
GBC093	South Korea	183	0·962	SBS1; SBS2; SBS7a; SBS13
GBC094	South Korea	34	0·813	SBS5; SBS7a; SBS13
GBC095	South Korea	35	0·719	SBS1; SBS6; SBS7b; SBS13
GBC096	South Korea	22	0·626	SBS5; SBS6; SBS10a; SBS10b
GBC097	South Korea	14	0·658	SBS1; SBS2; SBS13; SBS42; SBS54
GBC098	South Korea	238	0·977	SBS1; SBS2; SBS7a; SBS13
GBC099	South Korea	59	0·775	SBS1; SBS5; SBS7a; SBS13; SBS15
GBC100	South Korea	37	0·857	SBS2; SBS5; SBS6; SBS13; SBS52
GBC101	South Korea	305	0·904	SBS1; SBS5; SBS14; SBS15; SBS46
GBC102	South Korea	5	0·556	SBS1; SBS58; SBS59
GBC104	South Korea	74	0·916	SBS1; SBS2; SBS5; SBS13
GBC105	South Korea	136	0·928	SBS1; SBS2; SBS5; SBS13
GBC106	South Korea	27	0·595	SBS1; SBS5; SBS42
GBC107	South Korea	414	0·975	SBS1; SBS2; SBS7a; SBS13
GBC108	South Korea	155	0·867	SBS1; SBS2; SBS5; SBS8; SBS13; SBS30
GBC109	South Korea	23	0·63	SBS1; SBS5; SBS6; SBS13
GBC110	South Korea	55	0·947	SBS1; SBS2; SBS5; SBS7a; SBS13
GBC111	South Korea	61	0·863	SBS1; SBS2; SBS5; SBS13

GBC112	South Korea	45	0·815	SBS1; SBS2; SBS5; SBS8; SBS13
GBC113	South Korea	100	0·947	SBS1; SBS2; SBS5; SBS7a; SBS13
GBC114	South Korea	56	0·838	SBS1; SBS2; SBS5; SBS13; SBS84
GBC115	South Korea	176	0·78	SBS5; SBS57; SBS85
GBC116	South Korea	9	0·695	SBS5; SBS6; SBS10a; SBS19
GBC117	South Korea	33	0·726	SBS1; SBS5; SBS10b; SBS23
GBC118	South Korea	22	0·619	SBS1; SBS5; SBS30
GBC119	South Korea	14	0·754	SBS1; SBS7a; SBS13; SBS15; SBS28; SBS54
GBC120	South Korea	44	0·769	SBS1; SBS2; SBS5; SBS15; SBS39
GBC121	South Korea	18	0·609	SBS5; SBS28; SBS42
GBC122	South Korea	32	0·87	SBS1; SBS2; SBS5; SBS13; SBS23
GBC123	South Korea	25	0·695	SBS1; SBS2; SBS42
GBC124	South Korea	77	0·79	SBS2; SBS5; SBS13; SBS23; SBS39; SBS52
GBC125	South Korea	1	0·685	SBS42
GBC126	South Korea	34	0·732	SBS1; SBS5; SBS13; SBS24
GBC127	South Korea	25	0·656	SBS1; SBS5; SBS15
GBC128	South Korea	33	0·875	SBS1; SBS2; SBS5; SBS10a; SBS10b; SBS13
GBC129	South Korea	25	0·676	SBS1; SBS5; SBS10b; SBS16
GBC130	South Korea	183	0·927	SBS1; SBS5; SBS15; SBS20; SBS21
GBC131	South Korea	675	0·979	SBS1; SBS2; SBS5; SBS13
GBC132	South Korea	35	0·851	SBS1; SBS7a; SBS10b; SBS13
GBC133	South Korea	59	0·663	SBS1; SBS5; SBS19; SBS29
GBC134	South Korea	49	0·877	SBS1; SBS2; SBS5; SBS10b; SBS13
GBC135	South Korea	72	0·87	SBS5; SBS7a; SBS13; SBS15; SBS29
GBC136	South Korea	38	0·797	SBS2; SBS5; SBS10a; SBS13; SBS58
GBC137	South Korea	11	0·603	SBS1; SBS10a; SBS39
GBC138	South Korea	40	0·774	SBS1; SBS6; SBS42; SBS58
GBC139	South Korea	64	0·815	SBS1; SBS2; SBS7a; SBS13; SBS25
GBC140	South Korea	50	0·698	SBS1; SBS13; SBS22; SBS42
GBC141	South Korea	32	0·683	SBS1; SBS2; SBS5; SBS54
GBC142	South Korea	66	0·86	SBS2; SBS5; SBS13; SBS24

GBC143	South Korea	57	0·841	SBS1; SBS5; SBS7a; SBS13; SBS56
GBC144	South Korea	607	0·94	SBS1; SBS5; SBS6; SBS54
GBC145	South Korea	2369	0·805	SBS1; SBS5; SBS46; SBS54
GBC146	South Korea	146	0·945	SBS1; SBS2; SBS7a; SBS13; SBS42
GBC147	South Korea	74	0·787	SBS1; SBS5; SBS24; SBS31
GBC148	South Korea	72	0·619	SBS1; SBS5; SBS22
GBC149	South Korea	64	0·848	SBS1; SBS2; SBS5; SBS10b; SBS13; SBS42
GBC150	South Korea	53	0·566	SBS1; SBS5; SBS24

Supplementary Table S4. Cohort-level summary of significant focal copy-number alteration peaks identified by GISTIC2.

Cohort	Samples, n	Number of significant AMP peaks	Number of significant DEL peaks	Most significant AMP peak (cytoband)	AMP q-value	Most significant DEL peak (cytoband)	DEL q-value
Chile	23	1	2	6p21.2	1·67 × 10–2	18q21.2	8·89 × 10–3
China	51	0	2	None	None	1p36.13	6·55 × 10–4
India	59	1	6	6p12.3	1·23 × 10–1	19p13.3	3·67 × 10–2
Japan	38	2	5	3q26.2	3·86 × 10–3	8p21.3	1·23 × 10–3
South Korea	91	8	11	19q13.11	3·08 × 10–2	19p13.3	2·64 × 10–7

AMP, amplification; DEL, deletion; None, no significant peak identified. Significant peaks were defined as GISTIC2 focal peaks with q < 0·25. The top peak refers to the most significant focal event within each cohort. q-values are GISTIC2 false discovery rate–adjusted.

Supplementary Table S5. Recurrent significant focal copy-number alteration peaks identified by GISTIC2 across cohorts.

Event type	Cytoband	Cohorts	Number of cohorts	Lowest q-value	Cohort with lowest q-value
DEL	19p13.3	India, Japan, South Korea	3	2·64 × 10–7	South Korea
DEL	5q22.1	India, South Korea	2	1·95 × 10–3	South Korea

DEL, deletion. Recurrent lesions were defined as significant GISTIC focal events identified in at least two cohorts (q < 0·25). Lowest q-value refers to the most significant GISTIC2 q value observed across cohorts. No amplification peak met the recurrence criterion of significance in at least two cohorts (q < 0·25). q-values are GISTIC2 false discovery rate–adjusted.

Supplementary Table S6. Copy-number profiles of tumours without detectable nonsynonymous somatic SNVs/indels.

Tumour ID	Cohort	FGA	FGA class	No· of chromosome-level events	Major chromosomal events	Panel-gene events
GBC010T	India	0·0000	flat/diploid	0	None	None
GBC015T	India	0·0000	flat/diploid	0	None	None
GBC037T	India	0·1030	high-FGA	1	Broad chrX loss (~154·6 Mb)	None
GBC041T	India	0·1890	high-FGA	4	Broad chr13 gain (~95·1 Mb) plus additional gains	None
GBC103T	India	0·1380	high-FGA	3	Broad chr13 gain (~95·1 Mb); chr22 homozygous deletion (~34·1 Mb)	None
GBC125T	India	0·0227	low-FGA	1	chr22 gain (~34·1 Mb)	None
GBC195T	India	0·2770	high-FGA	6	chr13 and chr14 gains; chr17, chr19, and chr20 losses	*ERBB2* loss; *CCNE1* loss
GBC204T	India	0·0000	flat/diploid	0	None	None
GBC219T	India	0·0000	flat/diploid	0	None	None

FGA, fraction of genome altered, derived from the FACETS frac_alt_bp metric estimated from tumour–normal whole-exome sequencing data restricted to the shared region. FGA class indicate the categorisation of tumours as flat/diploid (FGA = 0), low-FGA (0 < FGA < 0·10), or high-FGA (FGA ≥ 0·10). Tumours listed here had no qualifying nonsynonymous somatic SNVs/indels in the harmonised consensus callset but were retained for copy-number analysis.

Supplementary Table S7. Distribution of tumour mutation burden (TMB) and the fraction of genome altered (FGA) according to tumour sample type.

Values are reported as median (IQR)

Metric	FFPE (n=13)	Fresh frozen (n=249)	p value (F-test)
TMB	1·28 (0·934–2·304)	2·50 (1·401–6·787)	0·0002
FGA	0·60 (0·354–0·750)	0·20 (0·086–0·495)	0·003

FFPE, formalin-fixed paraffin-embedded; IQR, interquartile range. p value, probability value from multivariable linear regression models testing differences by study cohort, age, sex, gallstones, tumour stage and the coverage in tumour and normal tissues as explanatory variables.

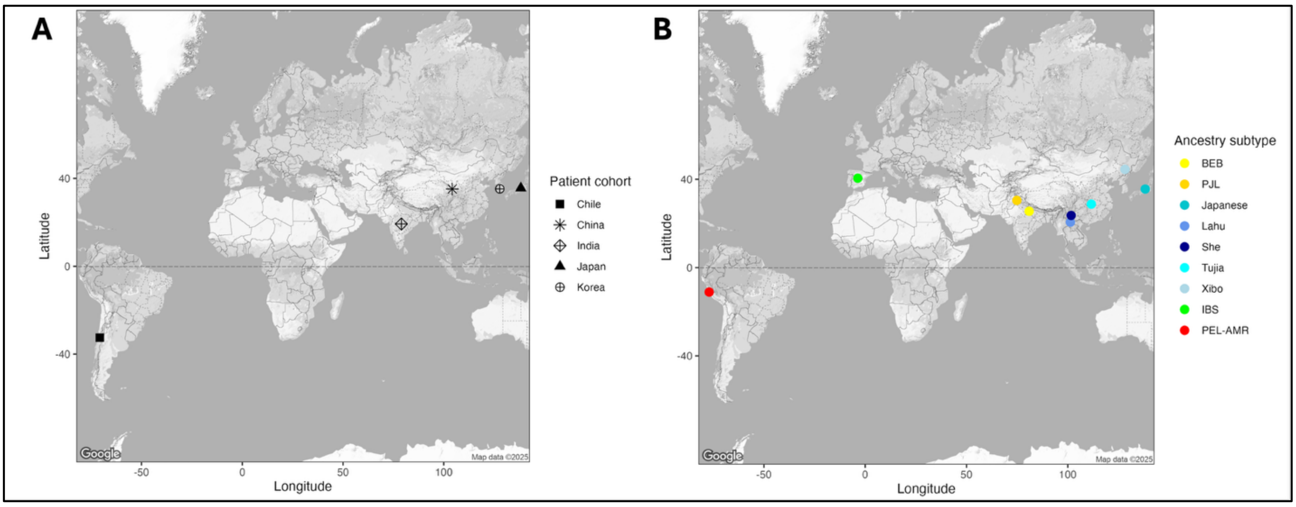

Supplementary Figure S1. Geographic location of the gallbladder cancer cohorts and the 1000 Genomes/HGDP reference populations used for ancestry inference.

A: Geographic location of the investigated cohorts of gallbladder cancer patients·
B: Geographic origin of the reference panels (populations) from the 1000 Genomes Project (1000GP) and the Human Genome Diversity Project (HGDP) used to estimate the proportions of genetic ancestry· BEB: Bengali in Bangladesh – 1000GP; PJL: Punjabi in Lahore – 1000GP; Japanese: Japanese population – HGDP; Lahu: Lahu population – HGDP; She: She population – HGDP; Tujia: Tujia population – HGDP; Xibo: Xibo population – HGDP; IBS: Iberian populations in Spain – 1000GP; PEL-AMR: Peruvian in Lima with estimated 100% Indigenous American ancestry – 1000GP.

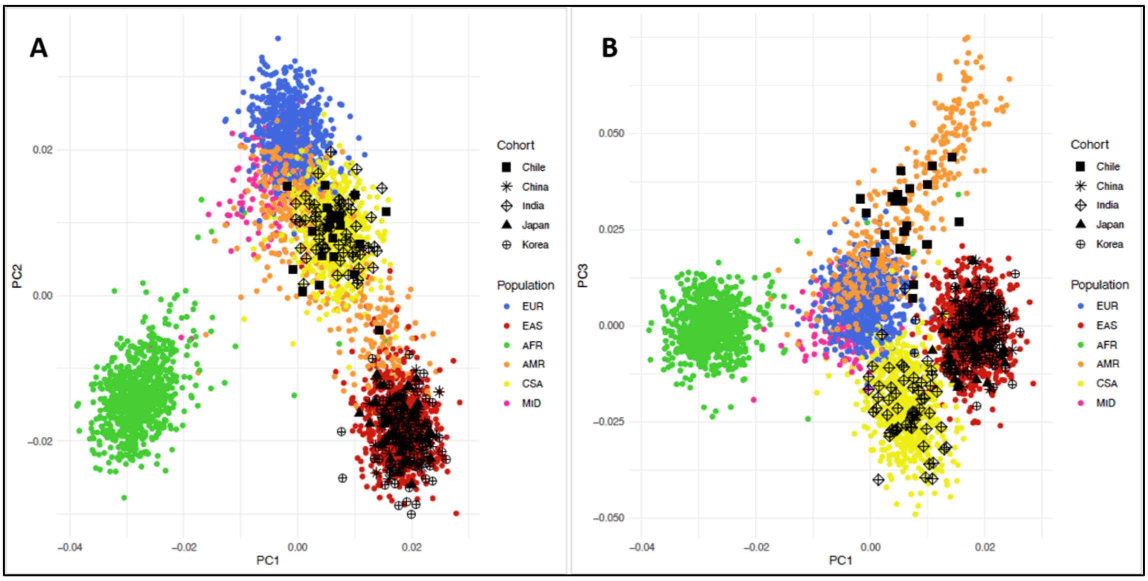

Supplementary Figure S2. Genetic principal component analysis of the five investigated cohorts of gallbladder cancer patients and the populations of the 1000 Genomes Project and the Human Genome Diversity Project.

EUR: European; EAS: East Asian; AFR: African; AMR: Admixed American; CSA: Central/South Asian; MID: Middle Eastern; PC: Principal component.

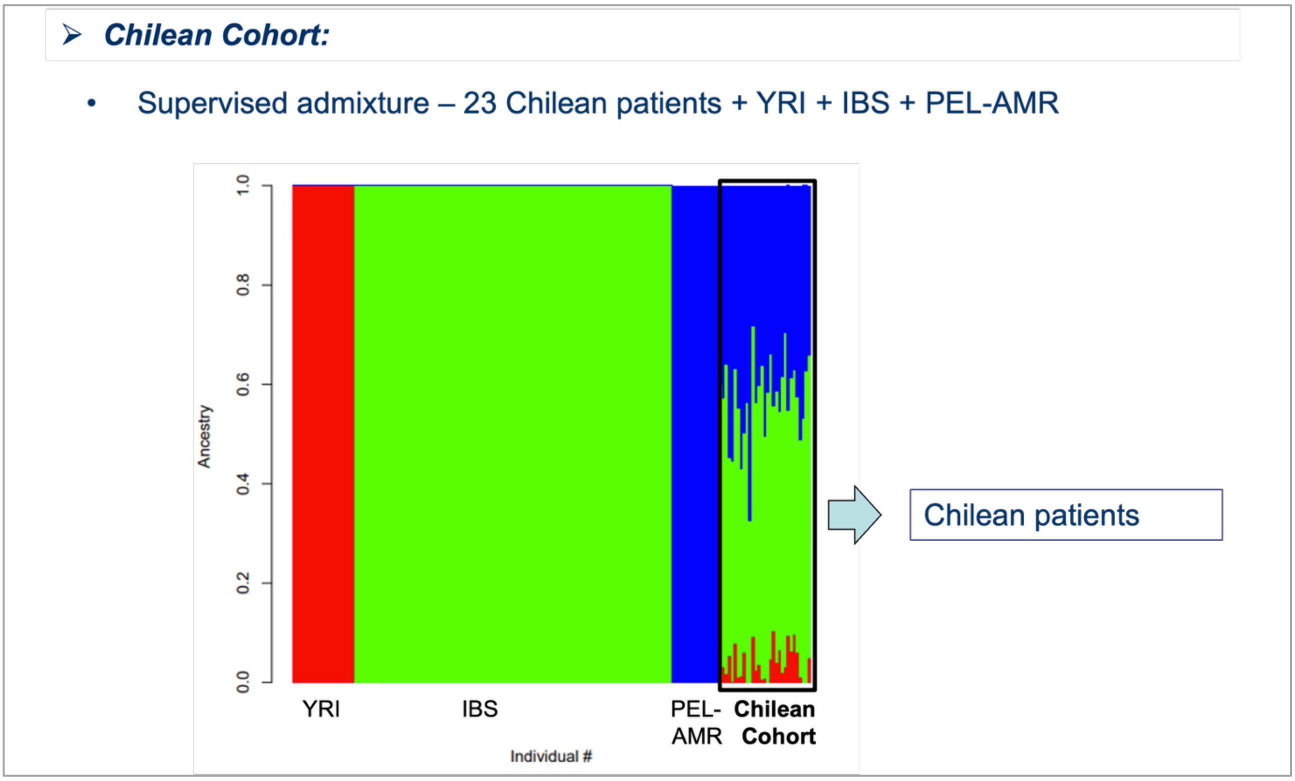

Supplementary Figure S3· Supervised ADMIXTURE ancestry estimates for Chilean gallbladder cancer patients using 1000 Genomes reference panels.

Supervised ADMIXTURE results for the 23 Chilean GBC patients using the Yoruba in Ibadan, Nigeria (YRI), the Iberian populations in Spain (IBS) and Peruvian in Lima with estimated 100% Indigenous American ancestry (PEL-AMR) from the 1000GP as reference ancestry panels.

A

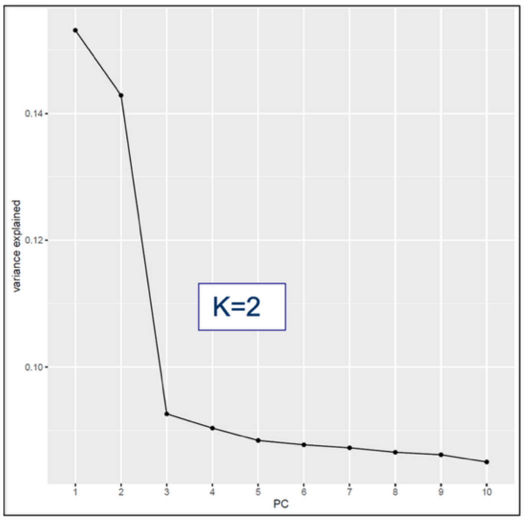

B

➢ ***Chilean Cohort :***

- Average proportions of ancestry subtypes

	Pop	Average
1	IBS	0,5340100
2	PEL-AMR	0,4303466

Supplementary Figure S4· Model selection and major ancestry subtypes in the Chilean cohort.
A: ADMIXTURE cross-validation plot for the Chilean cohort; B: major ancestry subtypes.

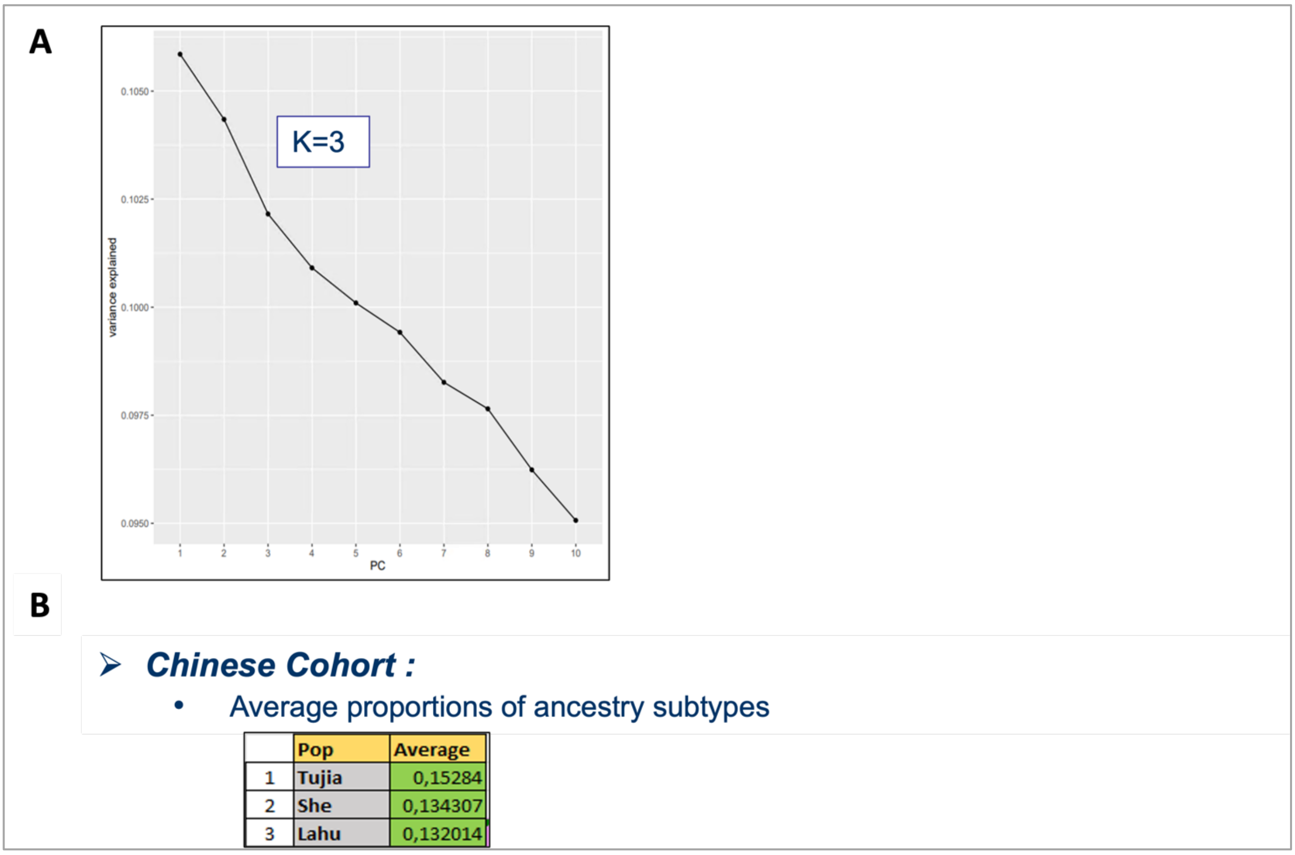

	Pop	Average
1	Tujia	0,15284
2	She	0,134307
3	Lahu	0,132014

Supplementary Figure S5· Model selection and major ancestry subtypes in the Chinese cohort.

A: ADMIXTURE cross-validation plot for the Chinese cohort; B: major ancestry subtypes.

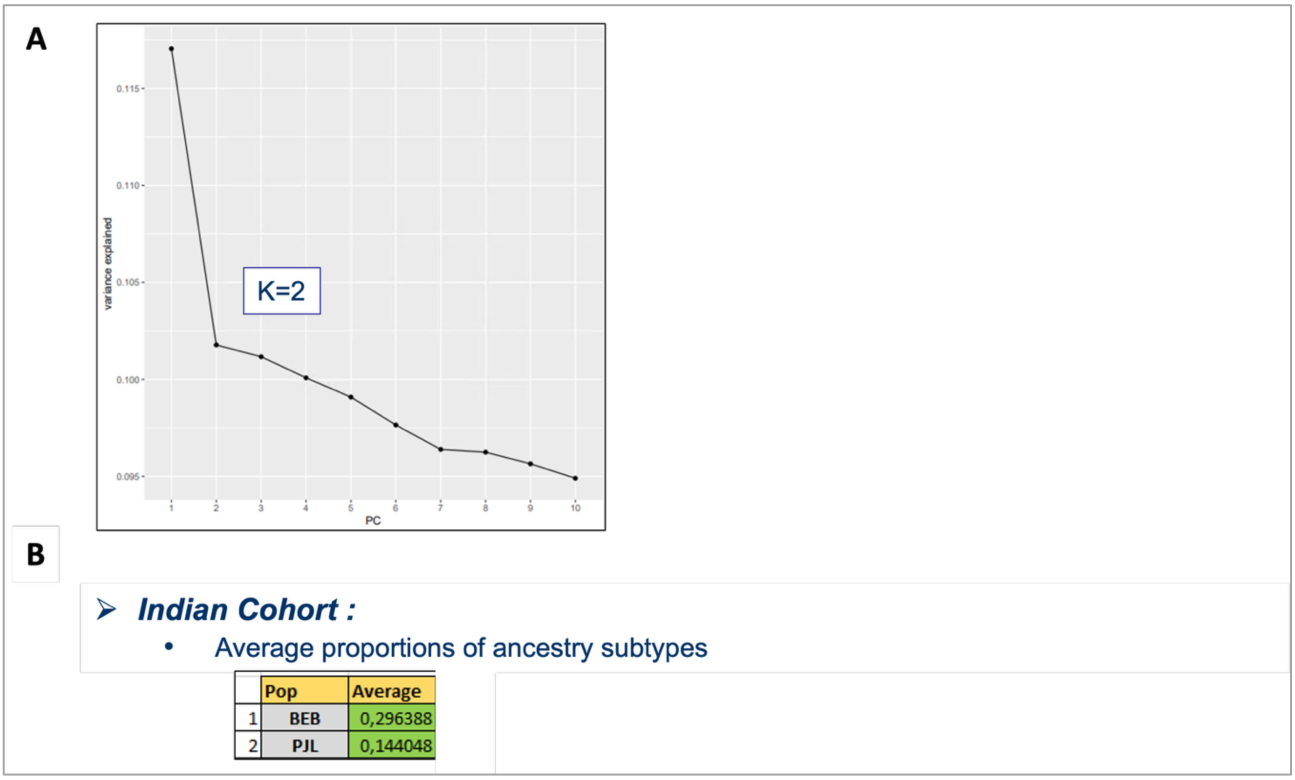

	Pop	Average
1	BEB	0,296388
2	PJL	0,144048

Supplementary Figure S6· Model selection and major ancestry subtypes in the Indian cohort.
A: ADMIXTURE cross-validation plot for the Indian cohort; B: major ancestry subtypes.

A

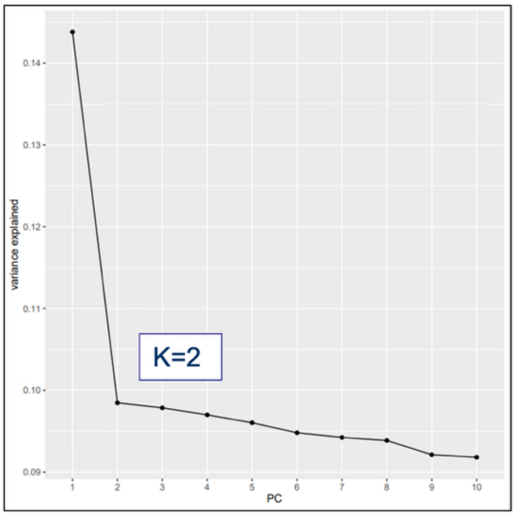

B

➢ ***Japanese Cohort :***

- Average proportions of ancestry subtypes

	Pop	Average
1	Lahu	0,739691
2	Japanese	0,157669

Supplementary Figure S7· Model selection and major ancestry subtypes in the Japanese cohort.
A: ADMIXTURE cross-validation plot for the Japanese cohort; B: major ancestry subtypes.

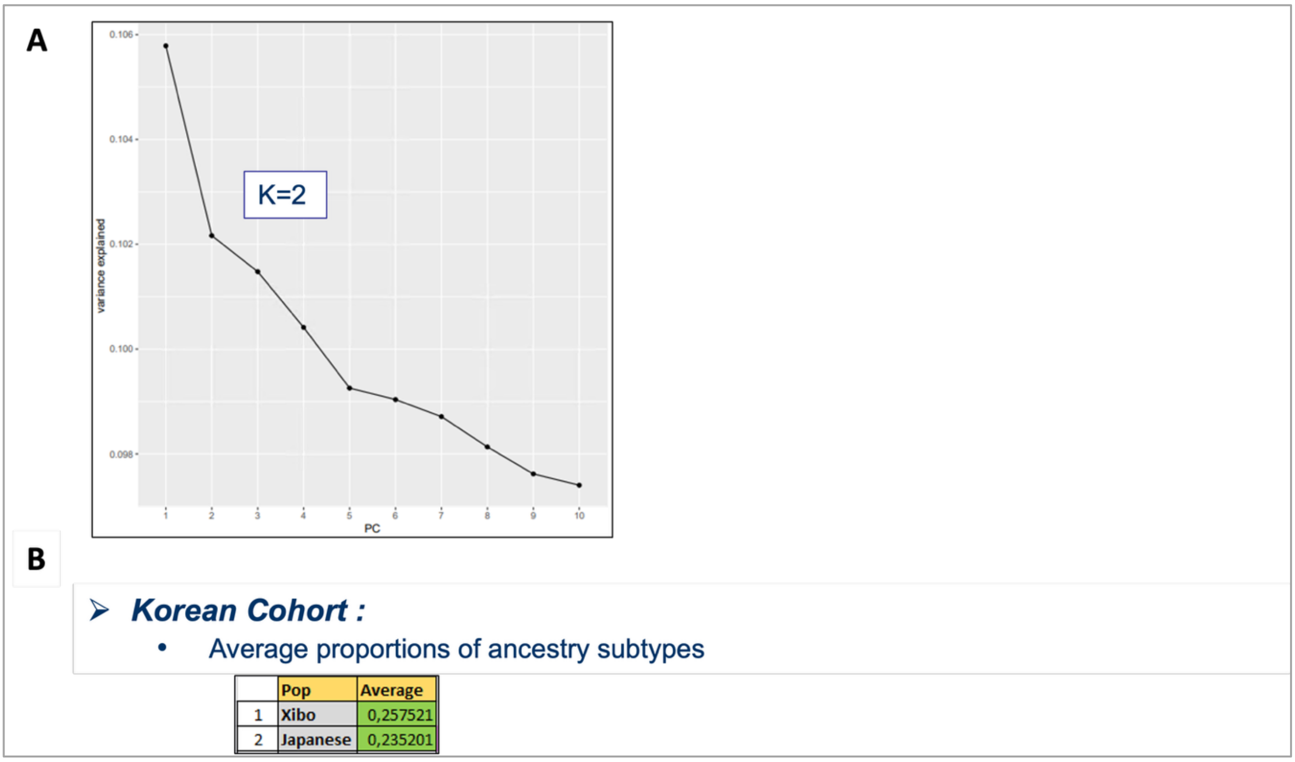

Supplementary Figure S8· Model selection and major ancestry subtypes in the South Korean cohort.

A: ADMIXTURE cross-validation plot for the South Korean cohort; B: major ancestry subtypes.

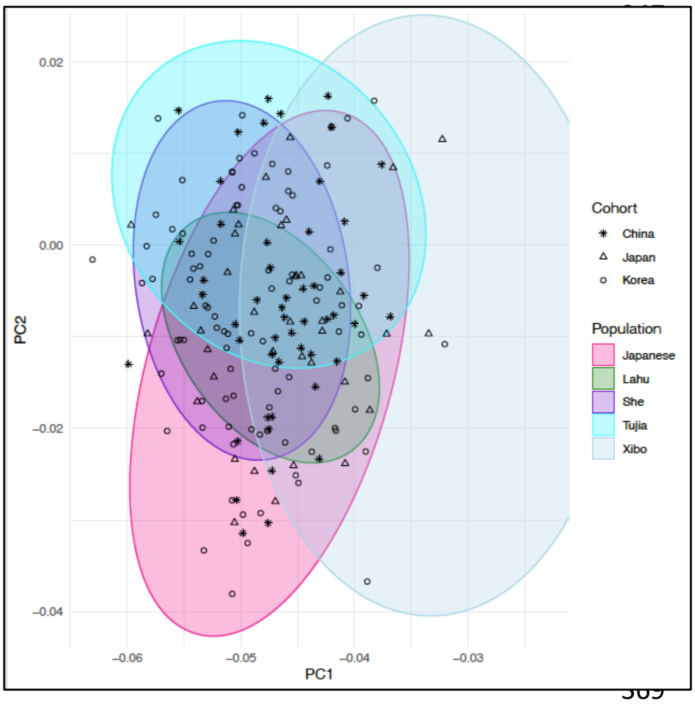

Supplementary Figure S9· Principal component analysis of Asian gallbladder cancer cohorts and HGDP reference panels for ancestry inference.

Genetic principal component analysis (PCA) of the three Asian cohorts of gallbladder cancer patients and the five reference panels used to estimate the proportions of genetic ancestry· Each ellipse represents a reference panel· Japanese: Japanese population – HGDP; Lahu: Lahu population – HGDP; She: She population – HGDP; Tujia: Tujia population – HGDP; Xibo: Xibo population – HGDP; PC1: first principal component; PC2: second principal components.

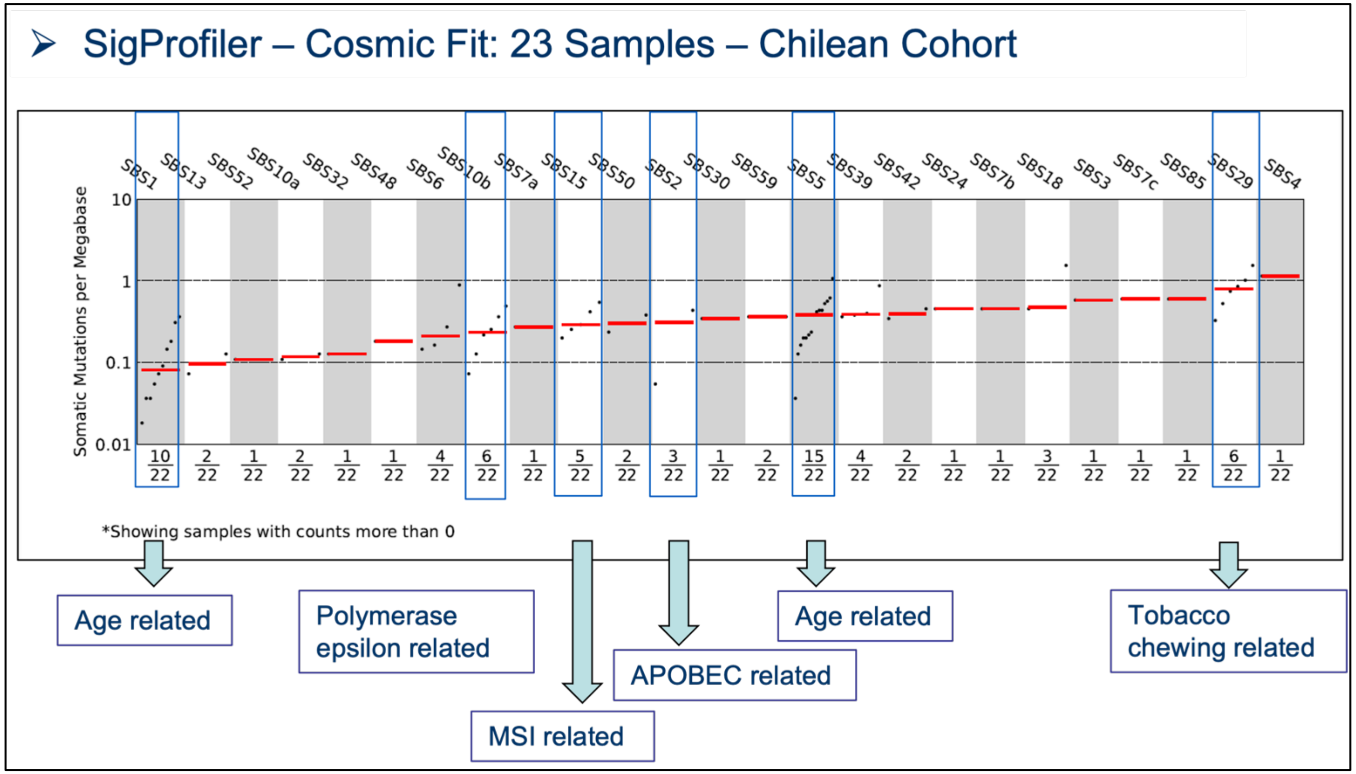

Supplementary Figure S10· Mutational signatures identified in the Chilean cohort.

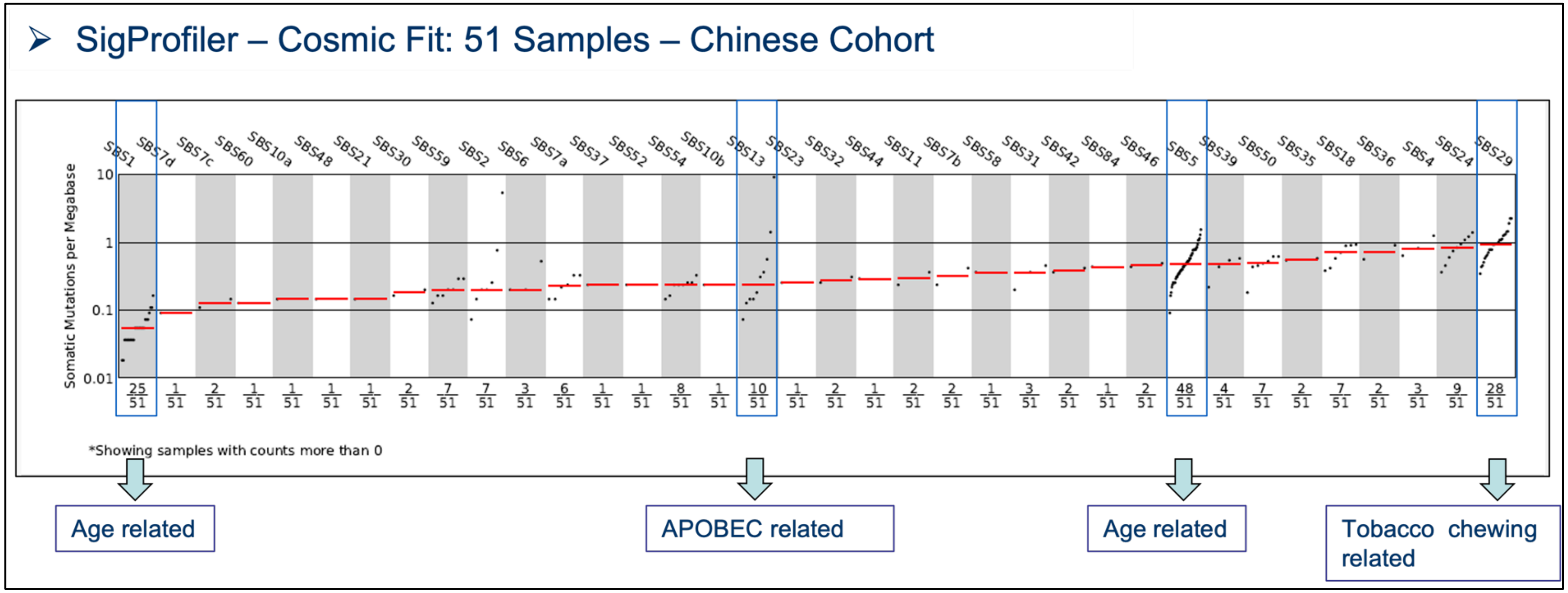

Supplementary Figure S11· Mutational signatures identified in the Chinese cohort

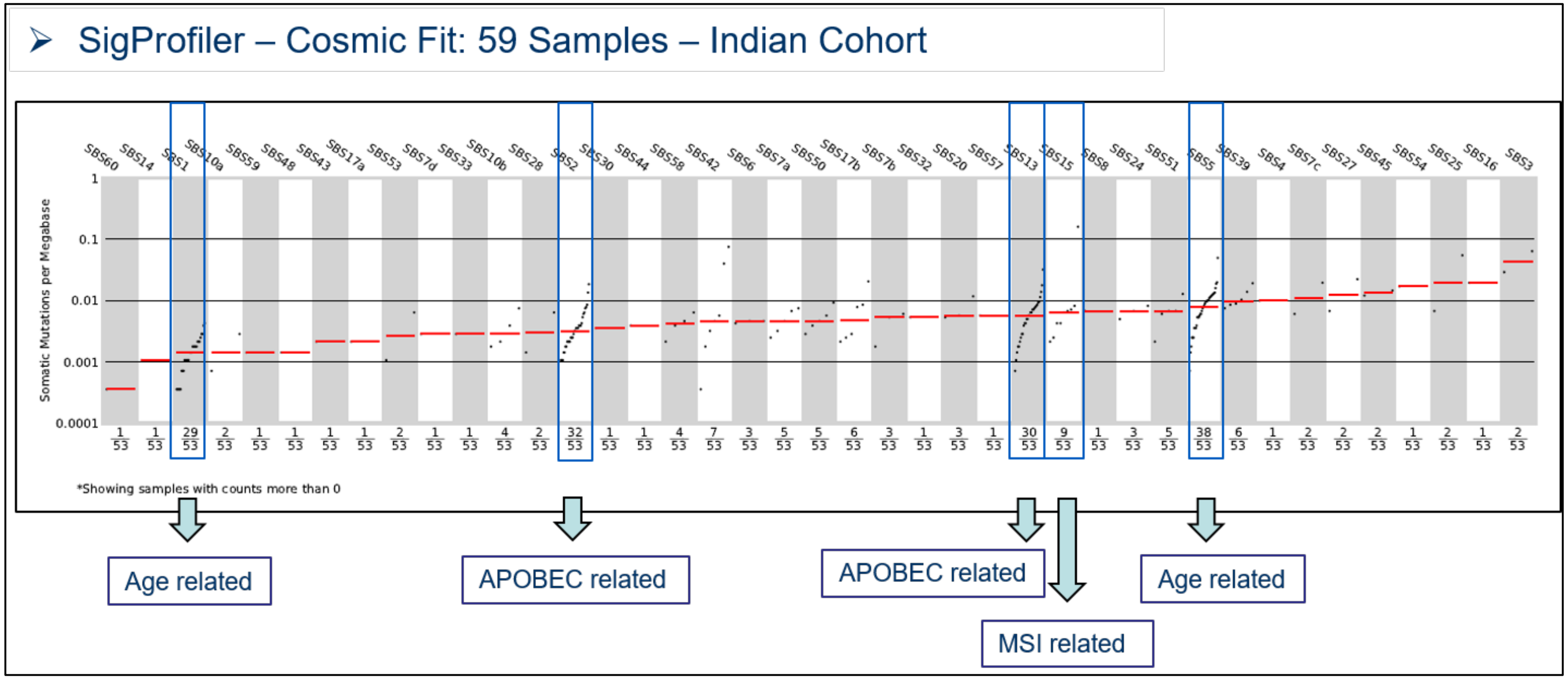

Supplementary Figure S12· Mutational signatures identified in the Indian cohort

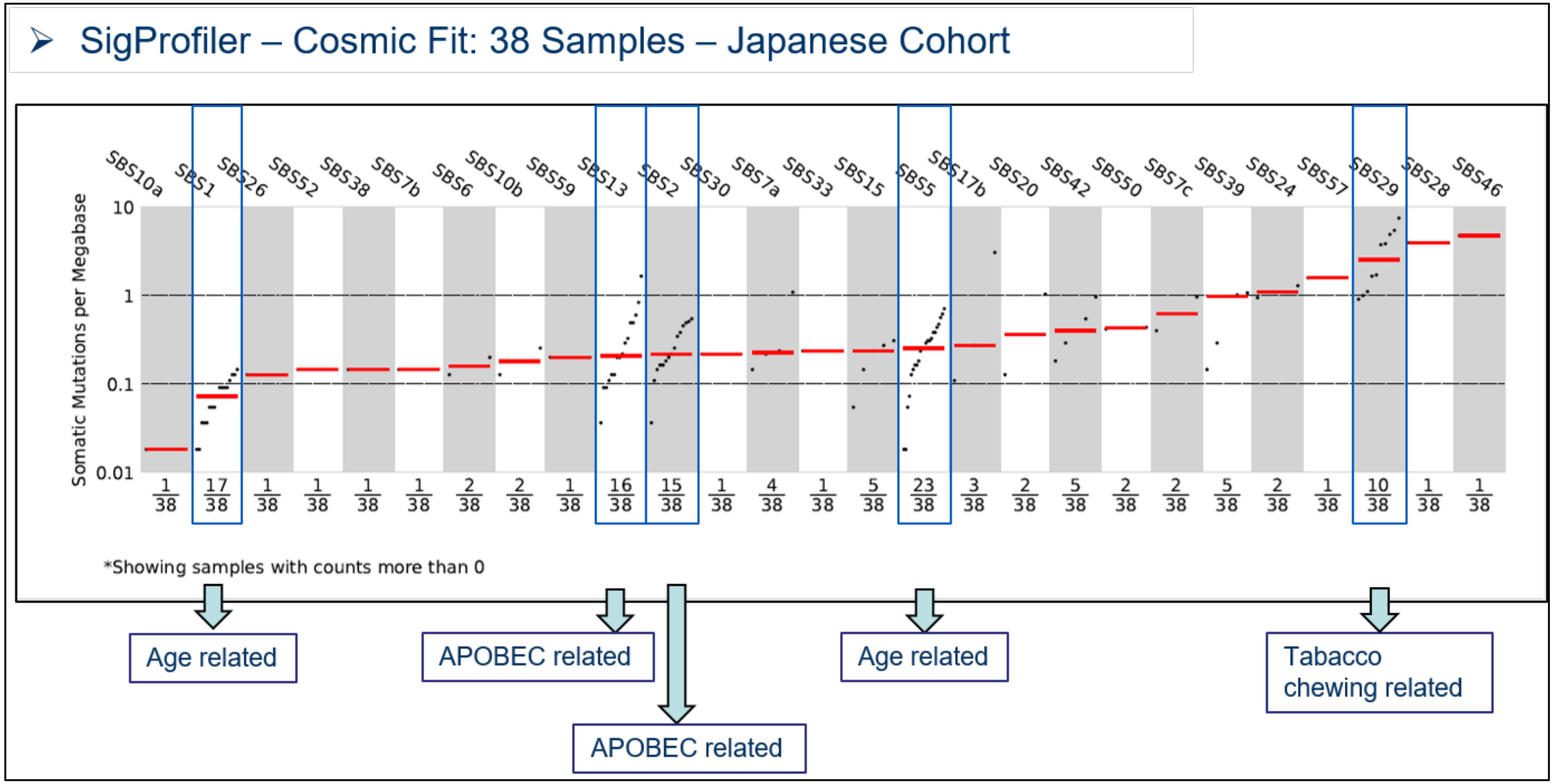

Supplementary Figure S13· Mutational signatures identified in the Japanese cohort

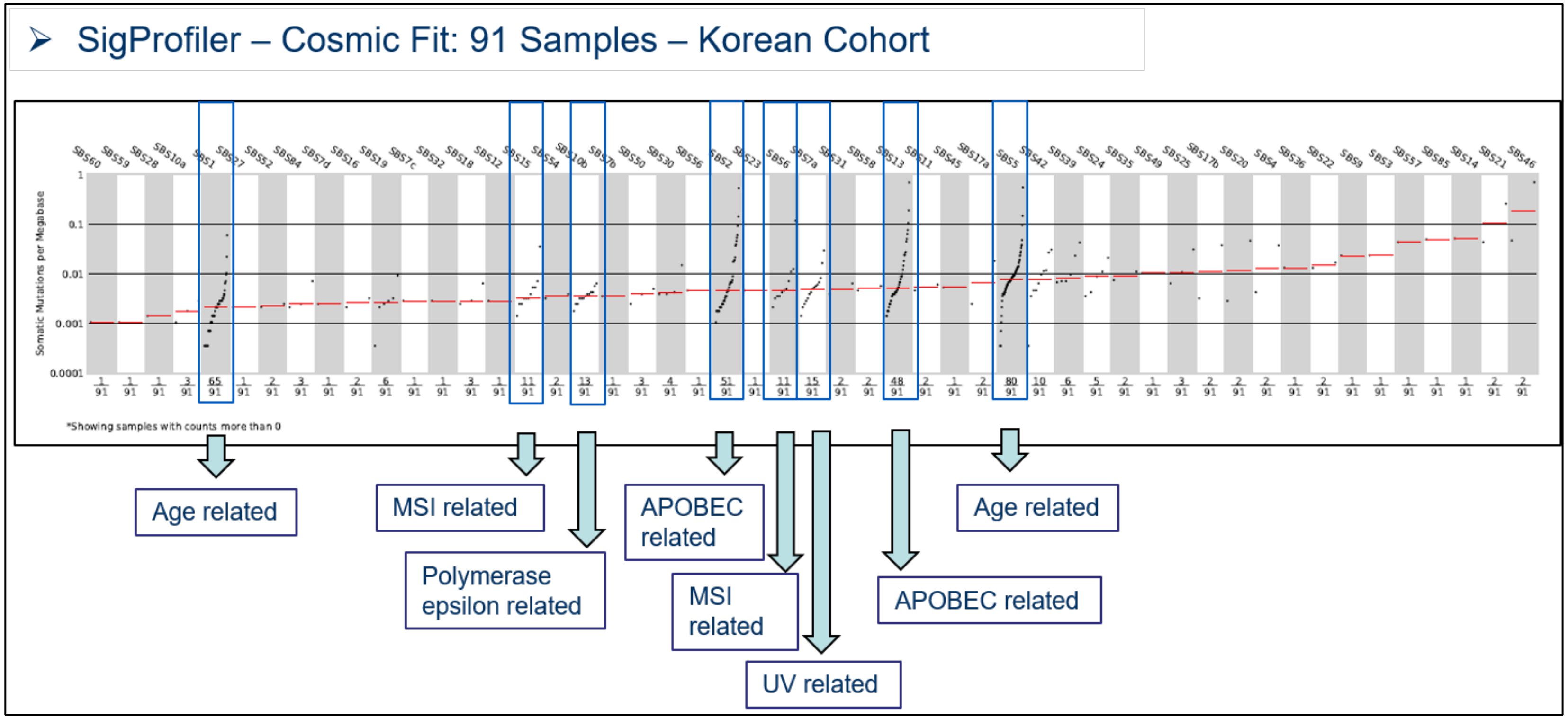

Supplementary Figure S14· Mutational signatures identified in the South Korean cohort.

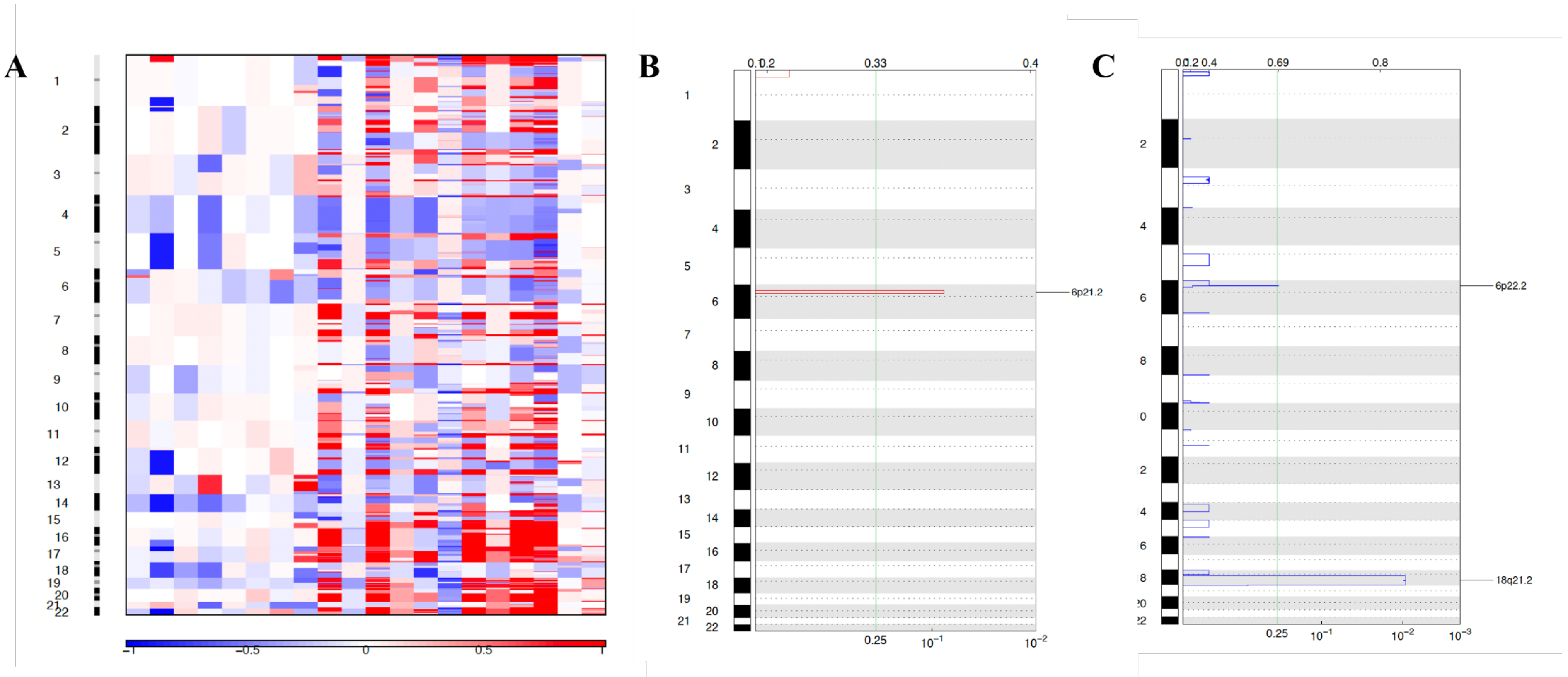

Supplementary Figure S15. Cohort-level copy-number profiles and GISTIC2 analysis in the Chilean cohort.

(A) Genome-wide FACETS-derived segmented copy-number heatmap of the Chilean cohort. Tumours are shown on the x-axis and genomic position along the y-axis. Colour intensity reflects the segment-level log2 copy-number ratio relative to the diploid state, with gains/amplifications shown in red and losses/deletions shown in blue. (B) GISTIC2 amplification profile showing significant focal amplification peaks. (C) GISTIC2 deletion profile showing significant focal deletion peaks. Significant GISTIC2 peaks were defined at $q < 0{\cdot}25$ after false discovery rate correction. Together, these panels summarise the sample-level copy-number distribution and recurrent cohort-level focal events identified by GISTIC2.

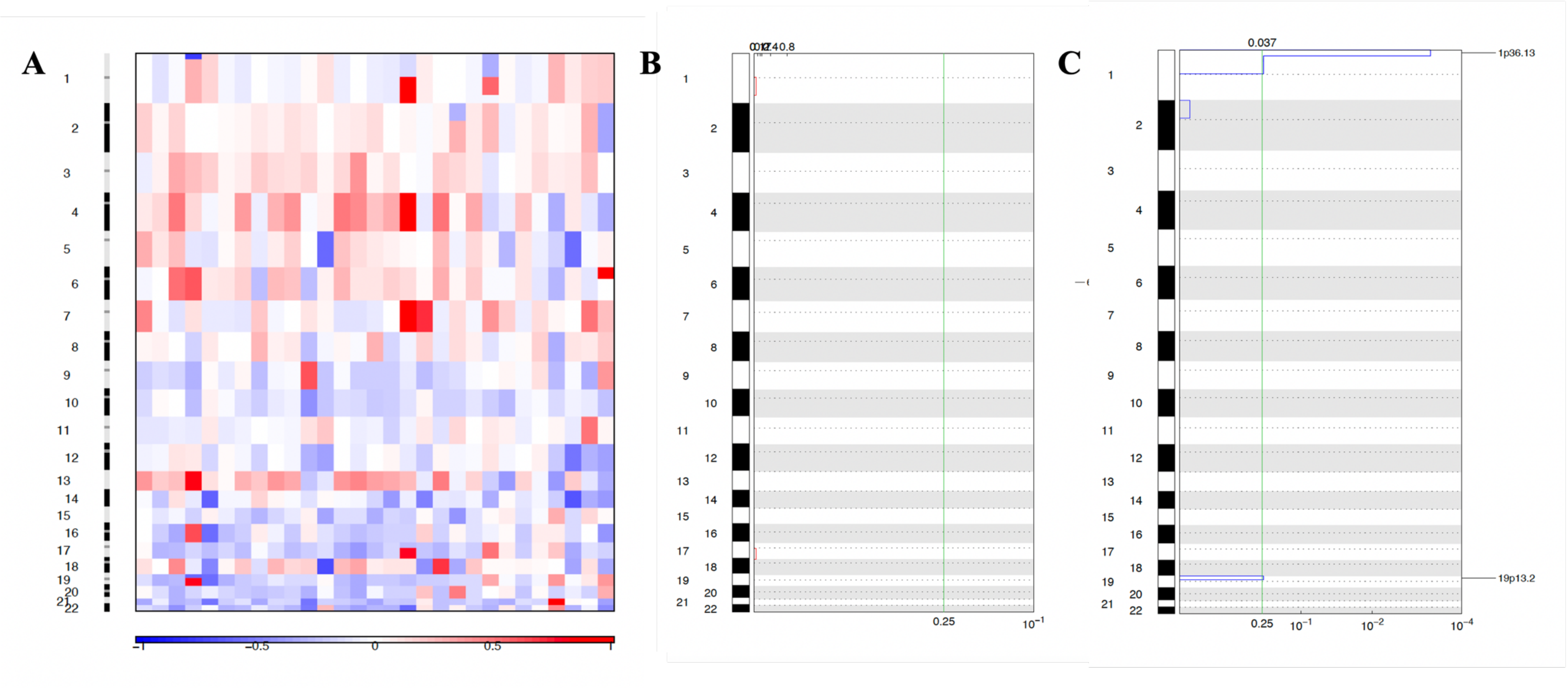

Supplementary Figure S16. Cohort-level copy-number profiles and GISTIC2 analysis in the Chinese cohort.

(A) Genome-wide FACETS-derived segmented copy-number heatmap of the Chinese cohort. Tumours are shown on the x-axis and genomic position along the y-axis. Colour intensity reflects the segment-level log2 copy-number ratio relative to the diploid state, with gains/amplifications shown in red and losses/deletions shown in blue. (B) GISTIC2 amplification profile showing significant focal amplification peaks. (C) GISTIC2 deletion profile showing significant focal deletion peaks. Significant GISTIC2 peaks were defined at $q < 0{\cdot}25$ after false discovery rate correction. Together, these panels summarise the sample-level copy-number distribution and recurrent cohort-level focal events identified by GISTIC2.

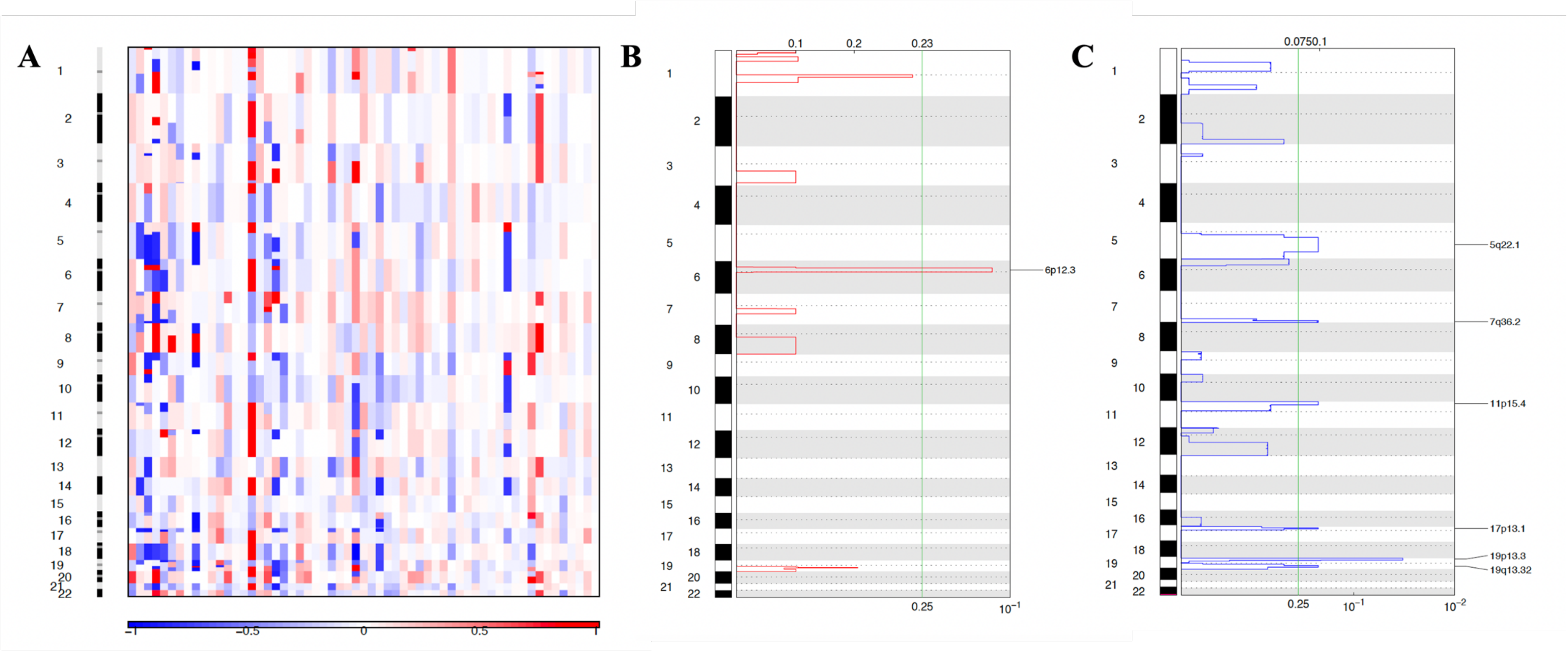

Supplementary Figure S17. Cohort-level copy-number profiles and GISTIC2 analysis in the Indian cohort.

(A) Genome-wide FACETS-derived segmented copy-number heatmap of the Indian cohort. Tumours are shown on the x-axis and genomic position along the y-axis. Colour intensity reflects the segment-level log2 copy-number ratio relative to the diploid state, with gains/amplifications shown in red and losses/deletions shown in blue. (B) GISTIC2 amplification profile showing significant focal amplification peaks. (C) GISTIC2 deletion profile showing significant focal deletion peaks. Significant GISTIC2 peaks were defined at $q < 0{\cdot}25$ after false discovery rate correction. Together, these panels summarise the sample-level copy-number distribution and recurrent cohort-level focal events identified by GISTIC2.

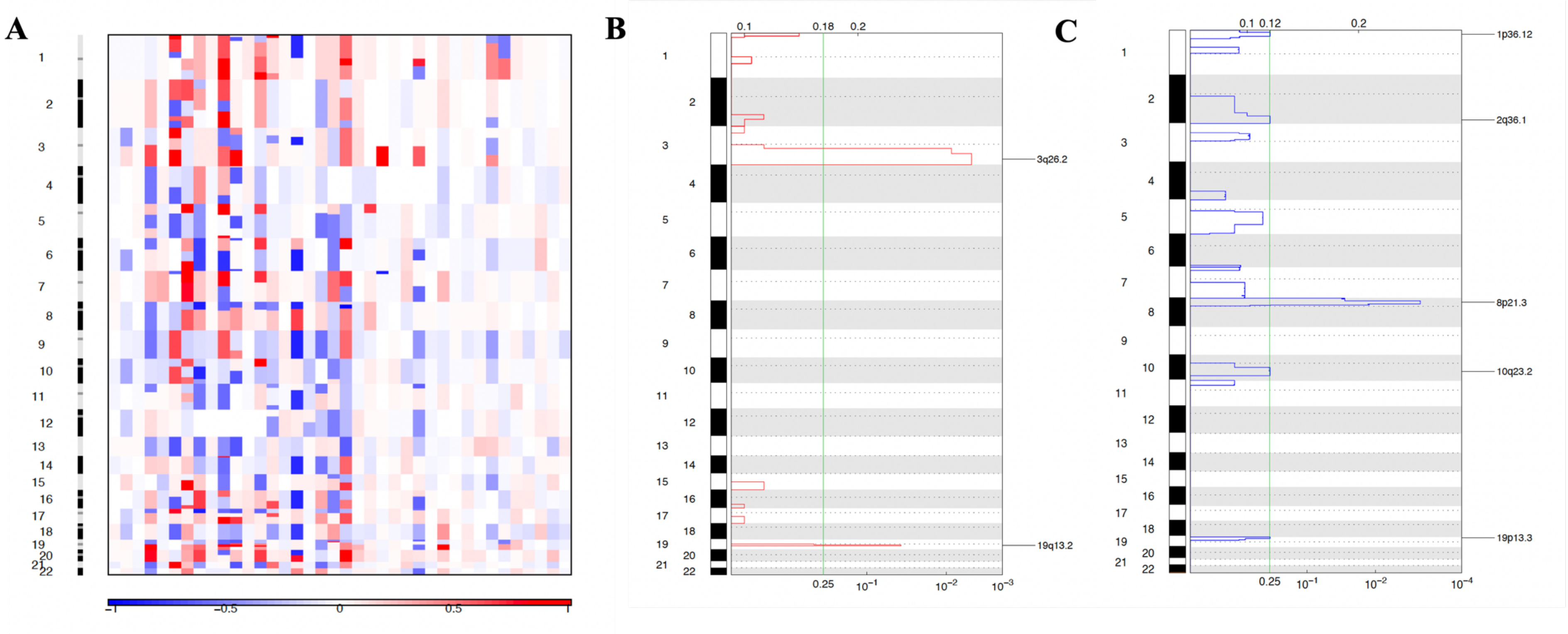

Supplementary Figure S18. Cohort-level copy-number profiles and GISTIC2 analysis in the Japanese cohort.

(A) Genome-wide FACETS-derived segmented copy-number heatmap of the Japanese cohort. Tumours are shown on the x-axis and genomic position along the y-axis. Colour intensity reflects the segment-level log2 copy-number ratio relative to the diploid state, with gains/amplifications shown in red and losses/deletions shown in blue. (B) GISTIC2 amplification profile showing significant focal amplification peaks. (C) GISTIC2 deletion profile showing significant focal deletion peaks. Significant GISTIC2 peaks were defined at q < 0·25 after false discovery rate correction. Together, these panels summarise the sample-level copy-number distribution and recurrent cohort-level focal events identified by GISTIC2.

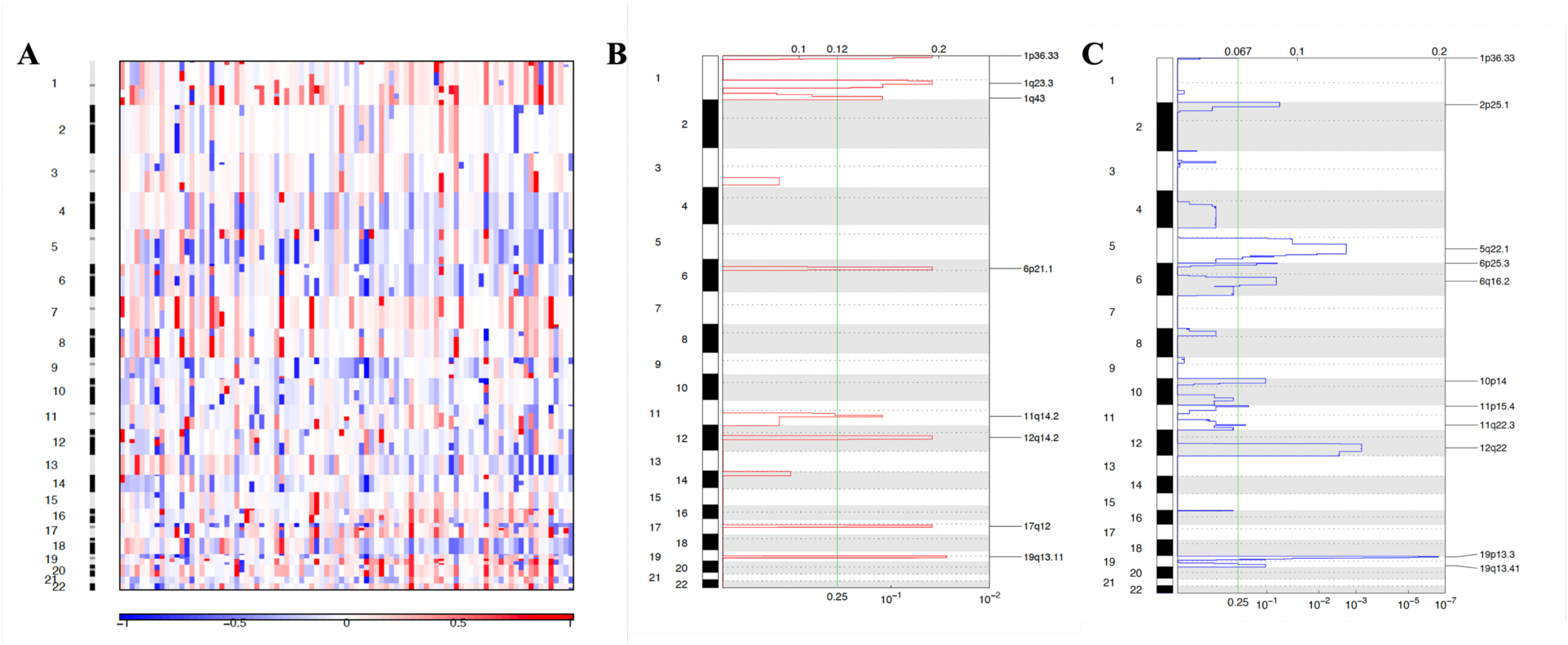

Supplementary Figure S19. Cohort-level copy-number profiles and GISTIC2 analysis in the South Korean cohort. (A) Genome-wide FACETS-derived segmented copy-number heatmap of the South Korean cohort. Tumours are shown on the x-axis and genomic position along the y-axis. Colour intensity reflects the segment-level log2 copy-number ratio relative to the diploid state, with gains/amplifications shown in red and losses/deletions shown in blue. (B) GISTIC2 amplification profile showing significant focal amplification peaks. (C) GISTIC2 deletion profile showing significant focal deletion peaks. Significant GISTIC2 peaks were defined at q < 0·25 after false discovery rate correction. Together, these panels summarise the sample-level copy-number distribution and recurrent cohort-level focal events identified by GISTIC2.

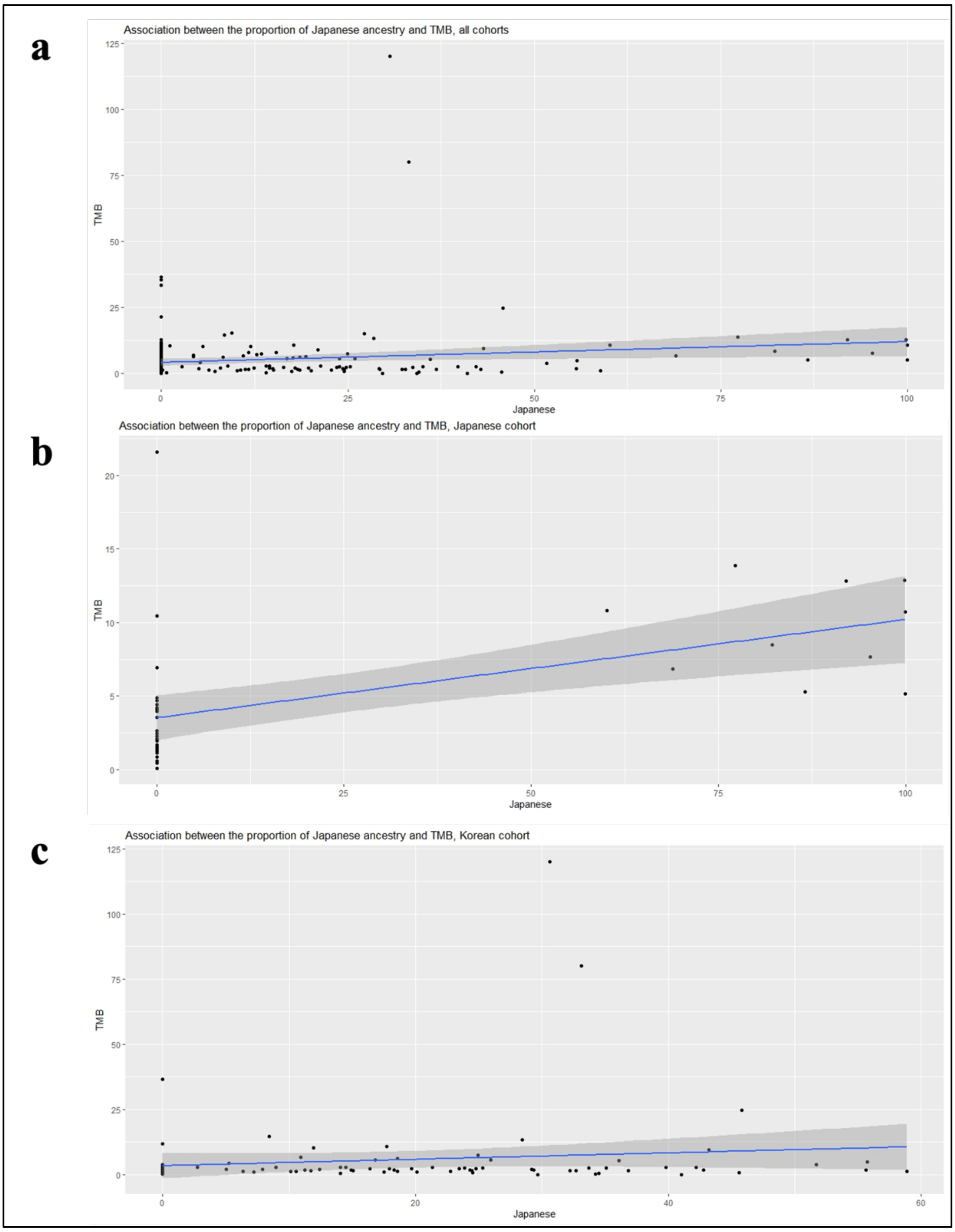

Supplementary Figure S20. Association between the proportion of Japanese ancestry and tumour mutational burden (TMB).

Scatter plots showing the relationship between the individual proportion of Japanese ancestry and TMB in (a) all cohorts combined, (b) the Japanese cohort, and (c) the Korean cohort. Each point represents one case. The blue line indicates the fitted linear regression line, and the shaded area represents the 95% confidence interval.

References

[1] Nakamura H, Arai Y, Totoki Y, Shirota T, Elzawahry A, Kato M, et al. Genomic spectra of biliary tract cancer. Nat Genet 2015;47:1003–10. https://doi.org/10.1038/ng.3375.
[2] Wardell CP, Fujita M, Yamada T, Simbolo M, Fassan M, Karlic R, et al. Genomic characterization of biliary tract cancers identifies driver genes and predisposing mutations. J Hepatol 2018;68:959–69. https://doi.org/10.1016/j.jhep.2018.01.009.
[3] Pandey A, Stawiski EW, Durinck S, Gowda H, Goldstein LD, Barbhuiya MA, et al. Integrated genomic analysis reveals mutated ELF3 as a potential gallbladder cancer vaccine candidate. Nat Commun 2020;11:4225. https://doi.org/10.1038/s41467-020-17880-4.
[4] Nepal C, Zhu B, O'Rourke CJ, Bhatt DK, Lee D, Song L, et al. Integrative molecular characterisation of gallbladder cancer reveals microenvironment-associated subtypes. J Hepatol 2021;74:1132–44. https://doi.org/10.1016/j.jhep.2020.11.033.
[5] Ebata N, Fujita M, Sasagawa S, Maejima K, Okawa Y, Hatanaka Y, et al. Molecular classification and tumor microenvironment characterization of gallbladder cancer by comprehensive genomic and transcriptomic analysis. Cancers 2021;13:733. https://doi.org/10.3390/cancers13040733.
